# Solubility of Incongruently Melting Active Pharmaceutical Ingredient Cocrystals: The Hydrochlorothiazide–Nicotinamide System

**DOI:** 10.1021/acs.molpharmaceut.5c01520

**Published:** 2026-02-10

**Authors:** Sahar Nasrallah, Tejas Gavali, Isil Yavuz, Mirjana Minceva

**Affiliations:** Biothermodynamics, TUM School of Life Sciences, 9184 Technical University of Munich, Maximus-von Imhof-Forum 2, Freising 85354, Germany

**Keywords:** drug solubility, solid−liquid equilibria, pharmaceutical cocrystals, eutectic mixtures, thermodynamic modeling

## Abstract

Pharmaceutical cocrystallization is a promising strategy to enhance the solubility and bioavailability of hydrophobic active pharmaceutical ingredients (APIs). However, when API–coformer cocrystals exhibit incongruent melting, understanding and predicting their solubility in water becomes significantly more complex. In this work, a combined experimental and thermodynamic modeling approach is presented to investigate the API solubility enhancement in a ternary API–coformer–water system. Hydrochlorothiazide (HCT), a biopharmaceutics classification system (BCS) class IV diuretic, and nicotinamide (Nic), a generally recognized as safe (GRAS)-listed coformer, were selected as a representative system that forms an incongruently melting 1:1 cocrystal, which was confirmed experimentally using differential scanning calorimetry (DSC) and powder X-ray diffraction (PXRD). Binary solid–liquid equilibrium (SLE) data for the HCT–Nic, HCT–water, and Nic–water systems were experimentally measured at different temperatures. The nonrandom two-liquid (NRTL) model was then used to regress the binary interaction parameters from the binary SLE data. These parameters were then used to predict ternary SLE phase diagrams of the HCT–Nic–water system at 310.15 K, 330.15 K, and 350.15 K. The NRTL-modeled SLE diagrams revealed the key features of the ternary system, including the absence of cocrystal formation at 310.15 K and the emergence of a cocrystal phase region with incongruent dissolution behavior at elevated temperatures. The highest HCT solubility was obtained at the ternary API-rich eutectic composition, with a solubility enhancement factor (Φ) of 2.1–2.4 across the studied temperatures. In contrast, dissolving the 1:1 cocrystal directly in water yielded significantly lower solubility enhancements (Φ ≈ 1.0–1.3). These findings clearly demonstrate that selecting a binary HCT–Nic mixture that, upon dilution in water, reaches the eutectic composition in the ternary HCT–Nic–water system yields greater solubility enhancement than starting from the cocrystal composition. This study emphasizes the importance of thermodynamic modeling in understanding solubility behavior and guiding the rational design of cocrystal-based pharmaceutical formulations, especially for API–coformer systems exhibiting incongruent melting.

## Graphical abstract

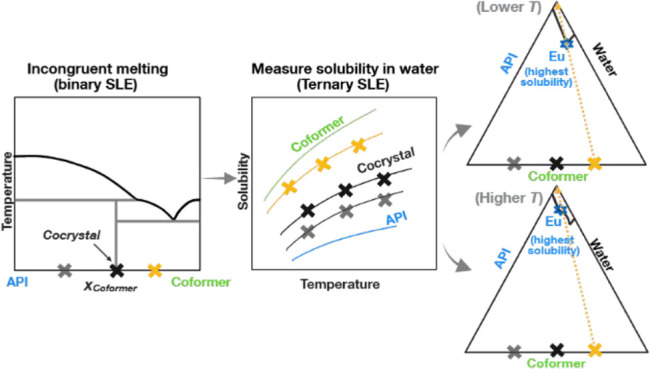

## 1 Introduction

Pharmaceutical cocrystals are multicomponent crystalline materials consisting of an API and a coformer, stabilized by noncovalent interactions such as hydrogen bonding, π–π stacking, or van der Waals forces.^1^ Over the past two decades, cocrystallization has emerged as a promising strategy to improve the aqueous solubility, dissolution rate, and bioavailability of poorly water-soluble APIs,^2^ thereby enhancing therapeutic efficacy and patient compliance.^3,4^ In addition to solubility enhancement,^5^ cocrystals can improve the physical^6^ and chemical^7^ stability of APIs, enhance tabletability,^8^ and modulate melting points to meet manufacturing requirements.^9^ Literature reports show that solubility enhancements achieved via cocrystallization span a wide range, from a few-fold^10–15^ to several tens-fold.^16^

Selecting an appropriate coformer is a critical step in cocrystal design, as it directly influences the resulting cocrystal’s physicochemical properties. The selection process commonly begins with computational prescreening methods that reduce the number of potential coformers to a manageable set before laboratory testing.^17^ These include hydrogen bond propensity (HBP) analysis, which predicts the likelihood of forming stable intermolecular interactions between the API and coformer;^18^ the ΔpK_a_ rule, which classifies systems as either cocrystals or salts based on acid–base strength differences between API and coformer;^19^ and thermodynamic models like conductor-like screening model for real solvents (COSMO-RS), which estimate the enthalpy of mixing to assess compatibility between the API and coformer (heteromeric interaction).^20^ Wang et al. developed a machine learning model trained on the entire cambridge structural database (CSD) to predict cocrystal formation using two-dimensional molecular structures of the components.^21^ Based on computational screening, a shortlist of pharmaceutically acceptable coformers (e.g., citric acid, saccharin, and urea) can be generated for each API as potential candidates for further experimental testing.^22^

Cocrystal stoichiometry is determined by the ability of the API and coformer to form noncovalent bonds and by the compatibility of their functional groups.^2^ The most common molar ratio is 1:1, as in the carbamazepine–nicotinamide cocrystal stabilized by N–H···N hydrogen bonds.^23^ The prevalence of 1:1 stoichiometry results from the tendency to maximize API–coformer interactions within the crystal lattice.^24^ Other ratios such as 2:1 or 1:2 can also occur when coformers possess multiple functional groups capable of forming more than one type of noncovalent interaction,^24^ such as caffeine-oxalic acid,^25^ theophylline-oxalic acid,^26^ and carbamazepine-succinic acid.^27^

To translate the potential benefits of cocrystals into functional formulations, a range of preparation methods has been developed, which differ in type of solvent use, energy input, and processing conditions. These methods are broadly categorized into solid-state, solution-based, and supercritical fluid methods.^28^ Solid-state methods include neat grinding (dry mixing of API and coformer) and liquid-assisted grinding (LAG), which uses a small amount of solvent to facilitate cocrystal formation. More advanced solid-state approaches, such as hot-melt extrusion, apply heat and mechanical shear to blend the API and coformer under controlled pressure. Solution-based methods—such as slurry conversion, evaporative or cooling crystallization, antisolvent addition, and spray drying—induce cocrystal formation by either supersaturation or solid–liquid equilibrium. Additionally, supercritical fluid-based methods, such as supercritical CO_2_-assisted slurry conversion and spray drying enable rapid crystallization under controlled thermodynamic conditions. Postsynthesis, the formation of a cocrystal is confirmed through analytical techniques such as PXRD to identify diffraction patterns distinct from those of the individual components and DSC to detect melting point shift relative to individual components.

According to the melting behavior, binary API–coformer cocrystals can be classified into two types: congruent and incongruent melting.^29^ In a congruent melting system, the cocrystal melts at a single temperature into a liquid with the same composition as the initial solid phase (the cocrystal), indicating phase equilibrium without decomposition.^30^ A classic example is the carbamazepine–nicotinamide (1:1 molar ratio) cocrystal, which exhibits a single sharp melting peak in the DSC thermogram, consistent with congruent melting behavior.^31^ In contrast, incongruent melting occurs when the cocrystal decomposes upon heating, producing a liquid phase and a different solid phase (typically the excess API or coformer), resulting in a liquid phase with a composition different from that of the original cocrystal.^1^ The carbamazepine–glutaric acid system demonstrates this behavior, in which the cocrystal melts incongruently, yielding a liquid solution and excess carbamazepine—as reflected by one sharp and one broad endothermic peak in the DSC thermogram.^32^ Understanding these melting behaviors is essential for interpreting SLE diagrams and predicting cocrystal stability and solubility during thermal processing, including drying, solvent evaporation, storage, or hot-melt extrusion.^33^

One of the main challenges in pharmaceutical cocrystal development lies not only in identifying suitable coformers but also in accurately predicting the solubility of the resulting cocrystals and selecting appropriate preparation methods.^30^ In particular, incongruently dissolving cocrystals in ternary API–coformer–solvent systems pose formulation difficulties, as conventional solvent-based techniques such as solution crystallization or solvent evaporation are often unsuitable.^32^ This is because the incongruently dissolving cocrystal is thermodynamically unstable along the stoichiometric composition line in the phase diagram, preventing its direct crystallization from solution.^1^ Incongruently dissolving cocrystals can be prepared using solid-state techniques such as LAG and slurry conversion. While LAG is effective for small-scale synthesis, slurry conversion is preferred for scale-up due to its compatibility for processing larger volumes of material.^2^ A thorough understanding of the SLE phase diagram in ternary API–coformer–solvent systems is therefore crucial, as it reveals the solubility of the cocrystal, the type of the crystallizing solid phase, and whether the system behaves congruently or incongruently.^1^ Knowledge of ternary SLE phase diagrams aids the selection of crystallization methods for the API–coformer cocrystals and determining the maximum API solubility in the solution at a specific temperature.

Experimentally determining the SLE phase diagram of a ternary system across different temperatures and compositions is labor-intensive and time-consuming.^34^ Alternatively, thermodynamic modeling allows prediction of SLE at any temperature and composition.^35^ Thermodynamic modeling requires knowledge of the melting properties of the pure components, the stoichiometry and melting properties of any existing cocrystal, and the activity coefficients of the API, coformer, and solvent in the liquid phase. Shortcut methods proposed by Rapeenun et al.^36^ and Perlovich^37^ estimate cocrystal solubility using simplified thermodynamic approaches that assume ideal solution behavior (activity coefficients equal 1). To reliably predict the complete SLE phase diagram of an API–coformer–solvent system, the temperature and composition dependence of activity coefficients must be accounted for.^34^ To predict the SLE of cocrystal systems while accounting for liquid solution nonideality, activity coefficient models such as the NRTL model^38^ can be employed.^39,40^ The NRTL model is a correlative model that utilizes experimental equilibrium-data to fit the model binary interaction parameters, without requiring molecular structural information.^38^ Building on our previous work^41^ with binary systems, this approach enables extension to ternary API–coformer–solvent systems.

Considering the increasing interest in pharmaceutical research on improving solubility through cocrystallization^1^ and the limited thermodynamic studies available for incongruently melting cocrystals, this study aims to investigate the aqueous solubility behavior of an incongruently melting API–coformer system using HCT–Nic system as a representative case. The objective of this study is to determine the binary HCT–Nic and ternary HCT–Nic–water SLE phase diagrams experimentally and to model the liquid-phase nonideality using the NRTL activity-coefficient model, enabling the identification of compositions that maximize the aqueous API solubility and providing insight into the temperature-dependent solid-phase stability region of the cocrystal in the ternary system. HCT a biopharmaceutics classification system (BCS) Class IV drug, commonly prescribed for hypertension,^42^ was selected as a representative API and Nic a generally recognized as safe (GRAS)-listed coformer^22^ as a representative coformer. The HCT–Nic cocrystal (1:1 molar ratio) is characterized by one-dimensional chains aligned parallel to the α-axis, formed through amide–pyridine synthons involving N–H···N hydrogen bonding.^42^ While the individual physicochemical properties of HCT, Nic, and their 1:1 incongruently melting cocrystal have been described in earlier studies,^42,43^ the complete SLE phase diagrams of the binary and ternary systems have not yet been investigated. This limited perspective may overlook alternative compositions in the HCT–Nic–water ternary system—such as eutectic mixtures at specific temperatures—where the API solubility is significantly enhanced compared to the cocrystal stoichiometry. Moreover, there is a lack of thermodynamic studies addressing how SLE behavior—particularly in incongruently melting systems—affects solubility at compositions beyond the cocrystal’s stoichiometry. To our knowledge, this is the first study to experimentally determine and thermodynamically model the complete binary and ternary SLE phase diagrams of an API–coformer system that forms a cocrystal with incongruent melting behavior, demonstrated here using the HCT–Nic system as a representative example with water as the solvent, providing new insight into the solubility behavior of this incongruently melting cocrystal beyond its stoichiometric composition. To address this gap, the present study conducts a comprehensive thermodynamic investigation of the binary and ternary systems through experimental measurements and thermodynamic modeling. The goal is to identify HCT–Nic compositions that maximize aqueous HCT solubility in solution and enable rational formulation design.

## 2 Materials and Methods

### 2.1 Chemicals

Nicotinamide (≥99%) and hydrochlorothiazide (≥99%) were purchased from Sigma-Aldrich (St. Louis, USA). HPLC-grade water was purchased from VWR Chemicals (VWR International, Radnor, USA). All chemicals were used as received without further purification. The molecular structures of the studied API and coformer are shown in Figure 1.

**Figure 1:**
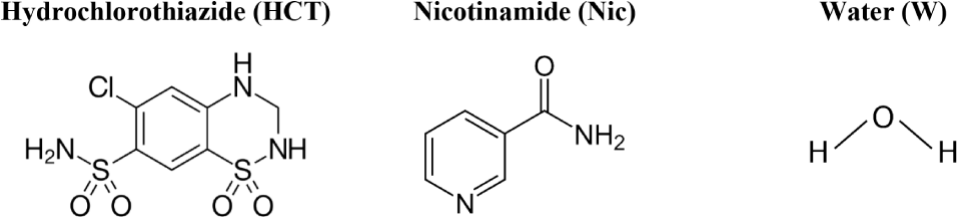
Molecular structures of the studied components.

### 2.2 Sample Preparation

Sample mixtures were prepared by weighing the required amounts of each component using a Sartorius analytical balance (Germany) with a precision of ±0.01 mg and transferring them into a mortar. Binary HCT–Nic mixtures were prepared in molar ratios ranging from 0.05:0.95 to 0.95:0.05. This range includes the equimolar 0.5:0.5 composition, which corresponds to the 1:1 cocrystal stoichiometric ratio. The components were manually ground with a pestle for 5 min to ensure homogeneity. Subsequently, 2–3 drops of methanol were added, and liquid-assisted grinding was continued for 15 min. The resulting paste was left to crystallize in a fume hood at room temperature for 5 days. To remove residual methanol, the samples were further dried in a drying incubator (myTemp 65, Benchmark) at 40 °C for 24 h. The dried samples were then used for subsequent analysis.

### 2.3 Powder X-ray Diffraction

PXRD was used to characterize the crystalline solid phases of the pure components (HCT and Nic) and their binary 1:1 cocrystal. Samples were packed into borosilicate glass capillaries (Hilgenberg, Germany) with a 1.5 mm diameter. PXRD measurements were performed at ambient temperature using Debye–Scherrer geometry on an STOE Stadi P diffractometer equipped with a DECTRIS Multi-MYTHEN detector, a curved Ge(111) monochromator, and a Mo Kα radiation source (λ = 0.70926 Å). Data were collected over a 2θ range of 2° to 39.55° with a step size of 0.015° (2θ). Two angular ranges were measured and summed to improve the signal-to-noise ratio. The phase purity of the purchased pure compounds was verified by comparing the measured PXRD patterns with calculated patterns derived from single-crystal X-ray or neutron diffraction data.^44^ The calculated PXRD patterns were generated using VESTA software.^45^ Minor deviations from the reference patterns were attributed to thermal expansion effects caused by differences in measurement temperature between literature data and the capillary PXRD experiments. The PXRD patterns of HCT and Nic, compared with calculated patterns, are shown in Figures S1 and S2, respectively, in the Supporting Information. Figures S1 and S2 show the PXRD patterns of the HCT and Nic starting materials, confirming the solid-state forms used in this study. The PXRD pattern of the HCT–Nic 1:1 cocrystal, along with those of the pure components at 303.15 K, are presented in Figure S3. To assess the cocrystal thermal stability and melting behavior, variable-temperature PXRD (VT-PXRD) of the cocrystal was performed at 303.15, 423.15, 443.15, 453.15, and 473.15 K. The diffraction patterns confirmed that the cocrystal remained as a single, stable solid phase up to 423.15 K, indicating congruent melting behavior. Above this temperature, the disappearance of characteristic peaks indicated cocrystal decomposition. The corresponding patterns are shown in Figure S4 of the Supporting Information.

### 2.4 Differential Scanning Calorimetry

DSC analysis was employed to determine the melting properties of the pure components and the SLE phase diagram of their binary system. The DSC measurements were performed using a NETZSCH DSC 200 F3 (Germany), calibrated with six reference standards (adamantane, indium, tin, zinc, bismuth, and cesium chloride) at a heating rate of 5 K min^–1^. All experiments were conducted under an inert nitrogen atmosphere with a flow rate of 150 mL min^–1^.

Samples of the pure compounds and binary mixtures were weighed using a Sartorius analytical balance (Germany) with a precision of ±0.01 mg. Binary mixtures were prepared in various molar ratios (0.05–0.95), as described in Section 2.2. For each mixture of HCT and Nic, approximately 6–8 mg of the ground powder was hermetically sealed in DSC pans and heated at a rate of 5 K min^–1^ to a maximum temperature of 540 K. This temperature was selected based on the melting points of the components to ensure complete melting without thermal degradation.

For the pure compounds, the melting temperature (*T_m_*) and melting enthalpy (Δ*h_m_*) were determined from the onset temperature and the integrated peak area, respectively. In the case of binary mixtures, the solidus and liquidus temperatures were identified as the onset of the first endothermic peak and the maximum of the second endothermic peak, respectively. The complete SLE data of the HCT–Nic binary system are provided in Table S1 (Supporting Information). The DSC thermograms of pure HCT, pure Nic, and the HCT–Nic cocrystal are shown in Figure S5 (Supporting Information), and the thermograms of their binary mixtures are presented in Figure 2b. In this study, only a single heating cycle was performed, as cooling and reheating have been found to induce polymorphic transformations of nicotinamide. All DSC measurements were performed in triplicate.

**Figure 2:**
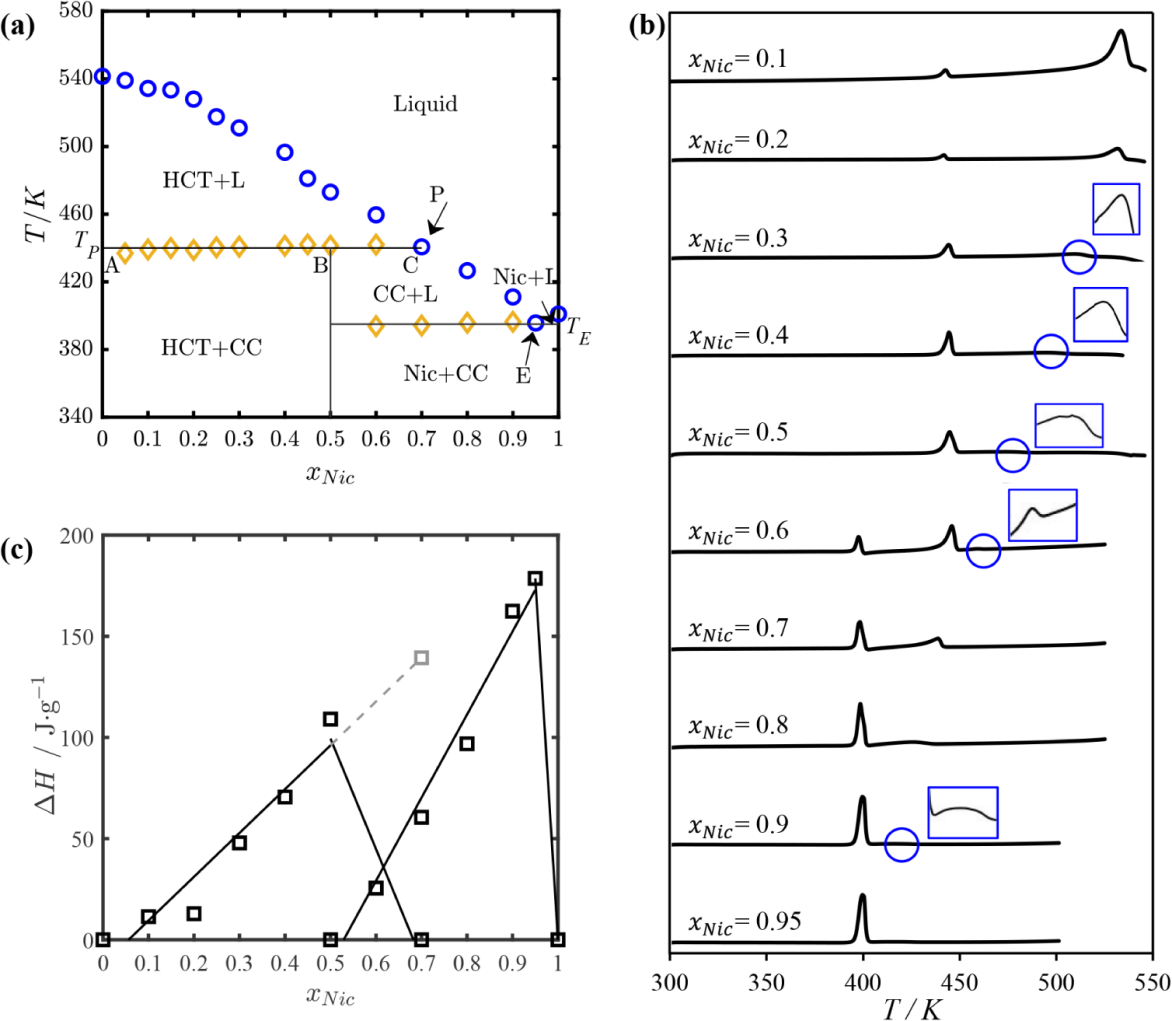
(a) Solid–liquid equilibrium phase diagram of the HCT–Nic system, showing incongruent melting behavior. Blue circles: experimental liquidus temperatures; orange diamonds: eutectic (solidus) temperatures; (b) DSC thermograms of HCT–Nic mixtures of different molar ratios including the cocrystal composition at *x_Nic_* = 0.5, with zoomed-in views provided to highlight small peaks; (c) Tammann plot for the HCT–Nic system.

### 2.5 Solubility Measurements

The solubility of pure HCT, Nic, and HCT–Nic binary mixtures with different molar ratios (0.05:0.90) in water was measured using a Crystal16 multireactor crystallizer (Technobis, Netherlands). This instrument determines the melting temperature of a solution by measuring light transmissivity through the sample, identifying the “clear point”—the temperature at which the solution becomes fully transparent at a known composition. Accurately weighed amounts of the solid phase were added to 1 mL of water in glass vials and then subjected to a thermal program under continuous stirring. The temperature was increased from 273.15 to 10 K below the boiling point of water at a rate of 0.5 K min^–1^. The clear point temperature was recorded as the liquidus temperature of the mixture at the given composition. All measurements were performed in triplicate and the precision of the clear-point measurements is reflected in the standard deviations of the triplicate measurements (between 0.02 and 1.14 K), which are reported in Table S2 of the Supporting Information. Preliminary measurements of the pH of representative HCT–Nic–water mixtures indicated neutral pH (≈7), with no significant variation expected under the dilute experimental conditions.

### 2.6 Thermodynamic Modeling

The solubility curves in the phase diagrams were calculated using

1 $$\ln x_{i}^{L}\gamma_{i}^{L}=-\frac{\Delta h_{m,i}}{RT}\left(1-\frac{T}{T_{m,i}}\right)-\frac{\Delta c_{p,i}}{R}\left(1-\frac{T_{m,i}}{T}+\ln\frac{T_{m,i}}{T}\right)$$

where $x_{i}^{L}$ and $\gamma_{i}^{L}$ are the mole fraction and activity coefficient of component i in the liquid phase, respectively; *T* is the temperature; Δ*h_m_*_,*i*_ and *T*_*m*,*i*_ are the melting enthalpy and melting temperature of pure component i , respectively; Δ*c*_*p*,*i*_ is the difference between the constant pressure heat capacity of pure component i in the solid and liquid states at *T*_*m*,*i*_; *R* is the universal gas constant. In many cases, the Δ*c*_*p*,*i*_ term (second term on the right-hand side) has a minor influence on the solubility curve compared to the Δ*h_m_*_,*i*_ term (first term on the right-hand side). Thus, for the sake of simplicity, Δ*c_p_*_,*i*_ term was not considered in this study, and the following expression was used to calculate the solubility line:

2 $$\ln x_{i}^{L}\gamma_{i}^{L}=-\frac{\Delta h_{m,i}}{RT}\left(1-\frac{T}{T_{m,i}}\right)$$

The cocrystal solubility line was calculated as follows:

3 $$\ln K=\ln K^{ref}+\frac{\Delta h^{ref}}{R}\left(\frac{1}{T^{ref}}-\frac{1}{T}\right)$$

4 $$K={\left(x_{A}^{L}\gamma_{A}^{L}\right)}^{v_{A}}{\left(x_{B}^{L}\gamma_{B}^{L}\right)}^{v_{B}}$$

where $v_{A}$ and $v_{B}$ are the stoichiometric coefficients of components A and B in the cocrystal. The cocrystal was selected as reference point, and accordingly, Δ*h^ref^* and *T^ref^* represent the melting enthalpy and melting temperature of the cocrystal, respectively. The reference solubility constant was calculated with the cocrystal stoichiometry and melting temperature as follows:

5 $$K^{ref}={\left(x_{ref,A}^{L}\gamma_{A}^{L}\left(x_{ref,A}^{L},T^{ref}\right)\right)}^{v_{A}}{\left(x_{ref,B}^{L}\gamma_{B}^{L}\left(x_{ref,B}^{L},T^{ref}\right)\right)}^{v_{B}}$$

6 $$x_{ref,A}^{L}=\frac{v_{A}}{v_{A}+v_{B}}$$

The activity coefficients of the components in the liquid phase were calculated using the NRTL model as follows:^38^

7 $$\ln\left(\gamma_{i}^{L}\right)=\frac{\overset{C}{\underset{j=1}{\sum }}\tau_{ji}G_{ji}x_{j}}{\overset{C}{\underset{j=1}{\sum }}G_{ji}x_{j}}+\overset{C}{\underset{j=1}{\sum }}\frac{x_{j}G_{ij}}{\overset{C}{\underset{k=1}{\sum }}x_{k}G_{kj}}\left(\tau_{ij}-\frac{\overset{C}{\underset{k=1}{\sum }}x_{k}\tau_{kj}G_{kj}}{\overset{C}{\underset{k=1}{\sum }}x_{k}G_{kj}}\right)$$

8 $$G_{ij}=\exp\left(-\alpha_{ji}\tau_{ij}\right)\;G_{ji}=\exp\left(-\alpha_{ji}\tau_{ji}\right)$$

9 $$\tau_{ij}=\frac{\Delta g_{ij}}{RT}\;\tau_{ji}=\frac{\Delta g_{ji}}{RT}$$

where Δ*g_i__j_* and Δ*g_j__i_* are the binary interaction parameters, and *α_ij_* is the nonrandomness factor, assumed equal 0.3. The binary interaction parameters were obtained by minimizing the following objective function:

10 $$\mathrm{OF}\left(T\right)=\overset{n}{\underset{i}{\sum }}{\left(\frac{{\left(T_{i}^{exp}-T_{i}^{cal}\right)}^{2}}{n}\right)}^{1/2}$$

where $T_{i}^{exp}$ and $T_{i}^{cal}$ are the experimental and calculated liquidus temperatures, respectively, and n is the number of data points. The discrepancy between the calculated liquidus temperatures $\left(T_{i}^{cal}\right)$ and the experimentally measured temperatures $\left(T_{i}^{exp}\right)$ was assessed by calculating the root-mean-square deviation (RMSD) as follows:

11 $$\mathrm{RMSD}=\sqrt{\frac{\overset{n}{\underset{i}{\sum }}{\left(T_{i}^{\exp}-T_{i}^{cal}\right)}^{2}}{n}}$$

### 2.7 Solubility Enhancement Factor

In both the binary API–water and the ternary API–coformer–water systems, the solubility (*S*) is defined as the ratio of the number of moles of API (*x*_1_) to the number of moles of water (*x*_3_) at a given temperature, for both binary systems (absence of coformer, *x*_2_ = 0) and ternary systems (presence of coformer, *x*_2_ ≠ 0):

12 $$S=\frac{x_{1}}{x_{3}}$$

where *x*_1_ and *x*_3_ are the mole fractions of API and water, respectively. The HCT solubility enhancement was evaluated using the solubility enhancement factor (Φ), which was defined as the ratio between the HCT solubility in the presence of the nicotinamide to its solubility in the absence of nicotinamide

13 $$\Phi =\frac{S_{x_{2}\neq 0}}{S_{x_{2}=0}}$$

where *x*_2_ is the nicotinamide (coformer) mole fraction.

The experimentally measured solubility of HCT–Nic mixtures with different molar ratios in water is provided in the Supporting Information (Table S2), where the data are expressed as mole fractions. To describe the temperature dependence of HCT solubility in the ternary API(1)–Nic(2)–water(3) system for each fixed HCT–Nic molar ratio, the experimental data were fitted using^46^

14 $$\ln x_{1}=A\left(T^{-1}\right)+B$$

where A and B are fitting parameters.

## 3 Results and Discussion

This section presents the experimental and thermodynamic modeling results for the HCT–Nic–water system. In Section 3.1, the experimentally measured binary and ternary SLE phase diagrams are presented. In Section 3.2, the binary and ternary SLE phase diagrams are modeled at different temperatures using the NRTL model, compared with the corresponding experimental data, and used to analyze the solubility enhancement of HCT in the ternary system.

### 3.1 Experimental Binary and Ternary SLE

#### 3.1.1 Binary SLE Phase Diagram

In Figure 2a, the experimentally determined SLE phase diagram of the HCT–Nic binary system is presented. Orange diamonds represent the eutectic (solidus) temperatures, and blue circles represent the experimentally measured liquidus temperatures. The corresponding DSC thermograms are presented in Figure 2b, with zoomed-in views highlighting small peaks. The onset temperature of the first endothermic event was taken as the solidus temperature (marked by orange diamonds in Figure 2a), while the maximum of the second peak was used to determine the liquidus temperature (marked by blue circles in Figure 2a). In the composition range 0 ≤ *x_Nic_* ≤ 0.5 and at temperatures below the solidus line, the system consists of excess HCT and the HCT–Nic 1:1 cocrystal (CC). Upon heating, the cocrystal decomposes at the peritectic temperature *T_p_* ≈ 447.15 K, followed by melting of the remaining HCT at the liquidus temperatures. The corresponding DSC thermograms (Figure 2b, 0 ≤ *x_Nic_* ≤ 0.5) exhibit a sharp first endothermic peak (cocrystal decomposition) and a broader second peak at higher temperatures (melting of excess HCT). At *x_Nic_* = 0.5, the DSC thermogram of the HCT–Nic 1:1 cocrystal (Figure 2b) exhibits two thermal events, indicating the incongruent melting behavior. The cocrystal decomposes at *T_p_* ≈ 447.15 K (first endothermic peak), during which solid HCT recrystallizes reversibly, while the liquid phase approaches the peritectic composition at *x_Nic_* = 0.7. Further heating leads to melting of the remaining HCT, producing the second endothermic peak. This behavior is typical for incongruently melting cocrystals, which decompose into a new solid and a liquid of different composition, rather than melting congruently into a homogeneous liquid that matches the stoichiometry of the cocrystal. In the composition range 0.5 ≤ *x_Nic_* ≤ 0.7, the binary HCT–Nic mixture contains excess nicotinamide and the HCT–Nic 1:1 cocrystal. Upon heating, the excess nicotinamide decomposes at the eutectic temperature *T_E_* ≈ 395.15K, followed by the incongruent melting of the cocrystal at the peritectic point *T_p_* ≈ 447.15 K, during which solid HCT recrystallizes reversibly. Further heating leads to the melting of the recrystallized HCT at the liquidus temperature. As shown in Figure 2b, the DSC thermogram at *x_Nic_* = 0.6 displays three distinct endothermic peaks: the first for eutectic melting of nicotinamide, the second for cocrystal decomposition, and the third for melting of the recrystallized HCT. In the composition range 0 ≤ *x_Nic_* ≤ 0.7, the liquidus line represents the HCT liquidus line.

In the composition range 0.7 ≤ *x_Nic_* ≤ 0.95, from the phase diagram presented in Figure 2a, it can be seen that the binary HCT–Nic mixture consists primarily of excess nicotinamide and a smaller amount of the HCT–Nic (1:1) cocrystal. Upon heating, the excess nicotinamide melts first at the eutectic temperature (*T_E_*), followed by incongruent melting of the cocrystal as the temperature approaches the cocrystal liquidus line. The corresponding DSC thermograms in Figure 2b display two main endothermic events: the first peak corresponds to the eutectic melting of excess nicotinamide, and the second to the decomposition of the cocrystal near the liquidus temperature. As the nicotinamide content increases, the second peak becomes less prominent, indicating a decreasing fraction of the cocrystal in the mixture. At *x_Nic_* = 0.95 (the eutectic point), the second endothermic peak disappears, indicating the complete absence of the cocrystal. In the range 0.7 ≤ *x_Nic_* ≤ 0.95, the liquidus line corresponds to cocrystal liquidus line, whereas in the range of 0.95 ≤ *x_Nic_* ≤ 1.0, it corresponds to the nicotinamide liquidus line, as shown in Figure 2a. The cocrystal and nicotinamide liquidus lines intersect at the eutectic temperature (*T_E_*), where the corresponding DSC thermogram in Figure 2b displays a single sharp peak.

In Figure 2c, the Tammann plot for the HCT–Nic system is shown, constructed by plotting the measured melting enthalpy (area under the first endothermic DSC peak) as a function of nicotinamide mole fraction. The first triangle 0 ≤ *x_Nic_* ≤ 0.7 corresponds to the incongruent (peritectic) melting of the cocrystal in the presence of excess HCT. The melting enthalpy is zero at *x_Nic_* = 0 (pure HCT, where no cocrystal is present), increases with nicotinamide content in the mixture as more HCT–Nic 1:1 cocrystal forms and decomposes upon heating, and reaches a maximum at *x_Nic_* = 0.5, where the cocrystal fraction is highest.

As the nicotinamide content increases further, the melting enthalpy decreases, approaching zero near the peritectic composition *x_Nic_* = 0.7, where the cocrystal phase has been entirely decomposed. The second triangle in Figure 2c (0.5 ≤ *x_Nic_* ≤ 1.0) corresponds to the eutectic melting of excess nicotinamide in the presence of the 1:1 HCT–Nic cocrystal. At *x_Nic_* = 0.5 (the cocrystal stoichiometry), no excess nicotinamide is present to undergo eutectic melting, and the corresponding melting enthalpy is 0. As the nicotinamide content in the mixture increases, the melting of the excess nicotinamide at the eutectic temperature becomes apparent, and the corresponding enthalpy increases. The enthalpy reaches a maximum near *x_Nic_* = 0.95, the eutectic composition. Beyond this point, as can be seen in Figure 2b, the eutectic peak vanishes, and at *x_Nic_* = 1, only a single endothermic peak corresponding to the melting of nicotinamide is observed.

Due to the incongruent melting behavior of the equimolar HCT–Nic 1:1 cocrystal, the first endothermic peak in the DSC thermogram (*x_Nic_* = 0.5, Figure 2b) reflects two simultaneous thermal events: cocrystal decomposition and reversible crystallization of HCT. Thus, the associated enthalpy cannot be solely attributed to the melting of the cocrystal. According to the lever rule, and assuming a total of 1 mol of 1:1 HCT–Nic cocrystal at *x_Nic_* = 0.5 and *T_p_*, the solid and liquid phase fractions, as well as the compositions of HCT and nicotinamide in each phase, can be estimated using the tie line in Figure 2a. The fraction of solid phase is given by BC/AC ≈ 0.29 and that of liquid phase by AB/AC ≈ 0.71, where the point B denotes the overall mixture composition (*x_Nic_* = 0.5), C is the peritectic liquid composition (*x_Nic_* = 0.7), and A is pure HCT (*x_Nic_* = 0). At *x_Nic_* = 0.5 the solid phase consists entirely of HCT, and the liquid phase composition matches the peritectic composition (*x_Nic_* = 0.7). Applying a mole balance, approximately 29% of the initial equimolar mixture converts to solid HCT, while 71% is liquid. As *x_Nic_* approaches 0.7, the lever arm BC shortens and becomes zero at the peritectic composition, indicating that no solid HCT crystallizes during cocrystal decomposition. Therefore, the enthalpy change associated with pure cocrystal decomposition was estimated by extending the linear segment of the Tammann plot up to *x_Nic_* = 0.7, as shown by the extended dashed blue line in Figure 2c. This approach is justified because at the peritectic composition, no solid HCT remains, and the cocrystal is the only phase undergoing decomposition.

#### 3.1.2 Ternary SLE Phase Diagram

In Figure 3, the experimental approach used to determine HCT solubility in the HCT–Nic–water ternary system is illustrated. The triangular diagram represents the full compositional space of the mixture. Blue triangles along the base of the diagram correspond to the initial binary solid mixtures of HCT and Nic with various molar ratios. To obtain a range of compositions along stoichiometric dilution lines (gray dashed lines with blue arrows in Figure 3) extending from the base toward the water apex, different amounts of the binary HCT–Nic solid mixtures with a fixed molar ratio were added to water. These dilution paths defined the experimental trajectories along which solubility measurements were performed. For each studied system composition along the dilution paths, the melting (liquidus) temperature was determined using the method described in Section 2.5.

**Figure 3:**
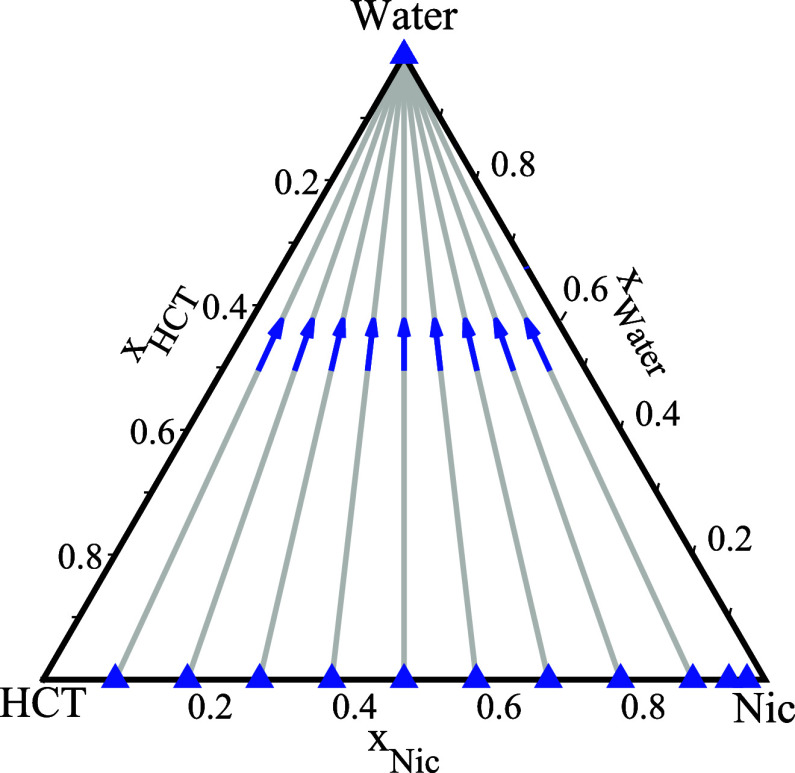
Composition paths in the ternary HCT–Nic–water system. Blue triangles on the base indicate fixed HCT–Nic molar ratios; blue arrows represent increasing water content along each stoichiometric dilution line toward the water apex.

In Figure 4, the experimental solubility of HCT (*x_HCT_*) in the HCT–Nic–water ternary system as a function of temperature, covering HCT:Nic molar ratios from 0.05:0.95 to 1.0:0.0 is presented. To improve clarity and avoid visual overcrowding, the data are presented in two graphs, both with the same *y*-axis scale. The low to moderate nicotinamide content (HCT:Nic molar ratios from 0.9:0.1 to 0.5:0.5) is displayed in Figure 4a, while the moderate to high nicotinamide content (HCT:Nic molar ratios from 0.4:0.6 to 0.05:0.95) is displayed in Figure 4b. The solubility measurements at the cocrystal stoichiometric composition (HCT:Nic = 0.5:0.5) in water are included in Figure 4a, and the corresponding numerical values are reported in Table S2 in the Supporting Information. At all investigated HCT:Nic molar ratios, HCT solubility increases with temperature. A slight improvement in solubility is observed with increasing nicotinamide content, particularly in Nic-rich mixtures (HCT:Nic = 0.05:0.95 in Figure 4b) at higher temperatures. However, the overlapping solubility curves indicate that the influence of nicotinamide on HCT solubility is relatively modest, becoming more noticeable only at higher HCT:Nic molar ratios.

**Figure 4:**
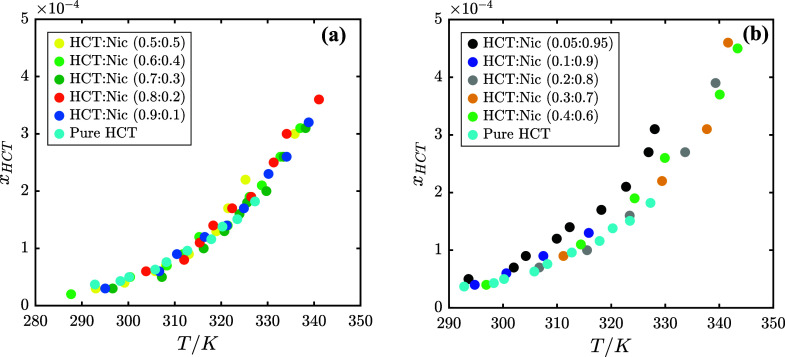
Experimental solubility of HCT in the HCT–Nic–water system at different HCT:Nic molar ratios as a function of temperature. (a) Low to moderate nicotinamide content (HCT:Nic molar ratios from 0.9:0.1 to 0.5:0.5); (b) Moderate to high nicotinamide content (HCT:Nic molar ratios from 0.4:0.6 to 0.05:0.95). In both graphs, the solubility of pure HCT in water (cyan circles) is shown for comparison.

The solubility data of HCT in the HCT–Nic–water system were further analyzed using the empirical correlation defined by eq 14. This equation was applied to each solubility curve shown in Figure 4 to fit the experimental data and determine the apparent thermodynamic parameters A and B, which are listed in Table S3 of the Supporting Information. In Figure 5, the natural logarithm of HCT solubility (In*x_HCT_*) as a function of inverse temperature (*T* ^–1^) for different HCT:Nic molar ratios is shown. It should be noted that a separate set of A and B parameters was determined for each HCT–Nic molar ratio. Results show a strong linear relationship between In­(*x_HCT_*) and *T* ^–1^, with coefficients of determination (*R* ^2^) greater than 0.99, confirming excellent agreement between experimental data and the empirical model.

**Figure 5:**
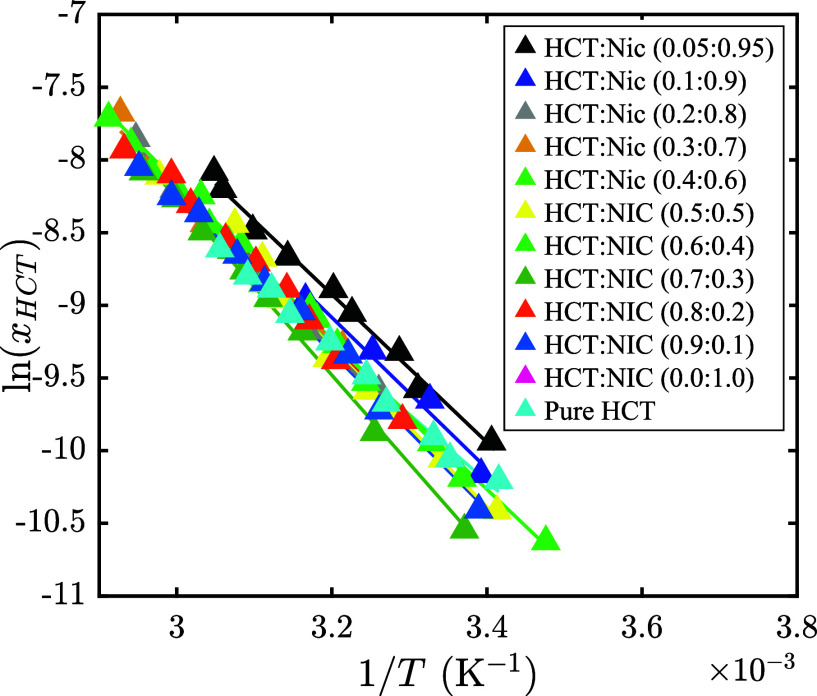
Natural logarithm of the HCT mole fraction In*x_HCT_* as a function of inverse temperature (*T* ^–1^) for various HCT:Nic molar ratios. Triangles: experimental data; lines: fitted eq 14 with A and B parameters in Table S3 of the Supporting Information.

The obtained correlations were used to calculate the SLE phase diagrams of the HCT–Nic–water ternary system at three different temperatures: 310.15, 330.15, and 350.15 K. For each fixed temperature, and for each HCT–Nic molar ratio used in the solubility experiments (ranging from 0 to 0.95), the corresponding HCT mole fraction in the ternary system (*x_HCT_*) was calculated using eq 14 and the fitted A and B parameters (Table S3 of the Supporting Information). The calculated ternary HCT–Nic–water composition (liquidus points) are presented in the ternary diagram in Figure 6 in mole fractions (blue circles). As can be seen in Figure 6, as the temperature increases from 310.15 to 350.15 K, the liquidus points (blue circles along the gray stoichiometry lines) shift downward toward the HCT–Nic side of the triangle, reflecting the expected increase in HCT solubility with temperature.

**Figure 6:**
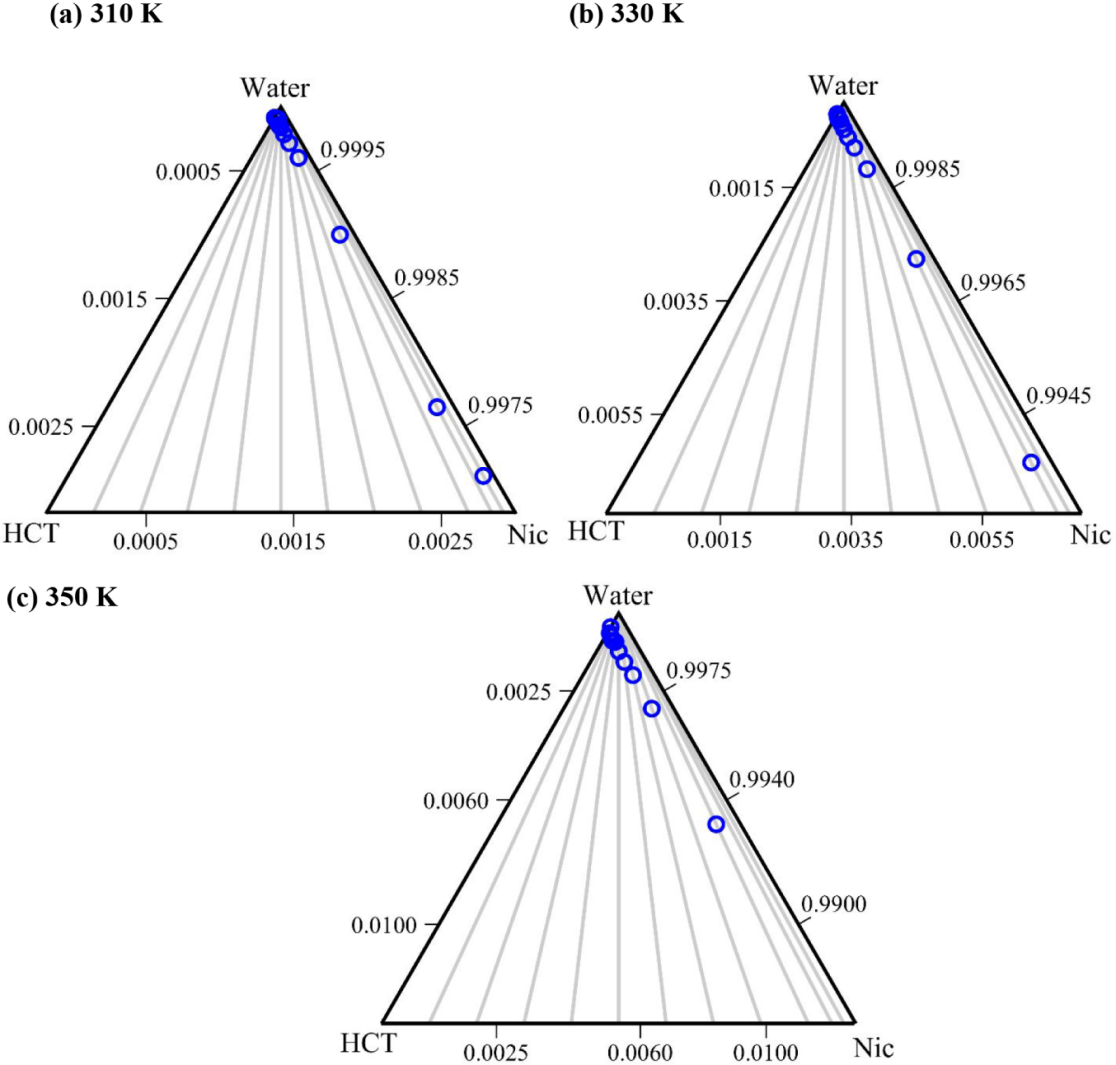
Zoomed-in view of the SLE phase diagrams of the HCT–Nic–water system in the dilute region. (a) 310.15 K; (b) 330.15 K; (c) 350.15 K. Blue circles: liquidus temperatures calculated using eq 14 and A, B parameters in Table S3 in Supporting Information; gray lines: stoichiometric HCT:Nic molar ratio lines.

### 3.2 Modeling Binary and Ternary SLE

The ternary SLE phase diagrams at different temperatures shown in Figure 6 provide valuable insight into the solubility behavior of HCT in the HCT–Nic–water system. However, the proposed approach for determining the isotherms becomes experimentally challenging at low HCT content (HCT:Nic ratios above 0.025:0.975), where accurately preparing very small compositional increments is required. For the system studied in this work, this limitation prevented the determination of isotherms in the low HCT concentration range. Additionally, the proposed method for determining the solubility isotherm does not reveal the type of solid phase present at equilibrium. Therefore, thermodynamic modeling is essential to fully describe the HCT–Nic–water SLE phase diagram. To model the ternary SLE phase diagram at various temperatures, the following must be known: (i) the melting properties of pure components, (ii) the melting properties and stoichiometry of the cocrystal, and (iii) the activity coefficients of each component in the liquid phase. In this study, the NRTL model was employed to model both the binary HCT–Nic and the ternary HCT–Nic–water phase diagrams, with melting properties from Table 1 and binary interaction parameters from Table 2. Table 1 summarizes the experimentally determined melting temperatures (*T_m_*) and melting enthalpies (Δ*h_m_*) of HCT, Nic, and their 1:1 cocrystal. The experimental melting properties were compared with literature data, showing excellent agreement. The melting enthalpy of the HCT–Nic cocrystal was estimated using the Tammann plot, as explained in Section 3.1.1.

**Table 1:** Melting Properties of the Studied Components, Measured in This Work and Compared to Literature Values

	*T_m_*/K		Δ*h_m_*/kJmol^–1^	
**Compound**	**This work**	**Literature**	**This work**	**Literature**
HCT	541.62 ± 0.49	540.75 ^47^	38.07 ± 0.89	37.60 ^48^
Nic	401.72 ± 0.06	401.60 ^48^	25.32 ± 0.25	25.50 ^48^
HCT–Nic 1:1 cocrystal	473.75 ± 0.76^b^	-	58.78^a^	-
Water	-	273.00 ^49^	-	6.01 ^49^

a For the cocrystals, the melting enthalpy was estimated using Tammann’s plot.

b For the incongruently melting HCT–Nic 1:1 cocrystal, the melting temperature (*T_m_*) was taken as the peak maximum of the second DSC endothermic peak (liquidus, complete melting). The onset temperature of the first DSC peak (*T_p_* = 443.75 K) corresponds to peritectic decomposition and is consistent with the value reported the literature (446.80 K).^42^

**Table 2:** NRTL Model Binary Interaction Parameters and Infinite Dilution Activity Coefficient ( $\ln\gamma_{1}^{\infty }$ and $\ln\gamma_{2}^{\infty })$ Calculated at 298.1 K^a^

**System (1–2)**	***g*_12_ (kJ mol** ^**–1**^)	***g*_21_ (kJ mol** ^**–1**^)	$\ln\gamma_{1}^{\infty }$ (−)	$\ln\gamma_{2}^{\infty }$ (−)	**RMSD (K)**
HCT–water	16.99	5.57	3.12	7.99	1.18
Nic–water^41^	7.79	–4.29	–0.51	0.23	1.94
HCT–Nic	–6.32	3.75	–3.97	–1.59	2.66

a The nonrandomness factor *α*_12_ = *α*_21_ = 0.3.

#### 3.2.1 Binary SLE Phase Diagram

The NRTL binary interaction parameters were obtained by fitting experimental SLE data of three binary systems: the HCT–Nic (Figure 2a), HCT–water (Figure 4), and the Nic–water system.^41^ In a binary system, the infinite dilution activity coefficient of the components ( $\ln\gamma_{1}^{\infty }$ and $\ln\gamma_{2}^{\infty }$ ) indicates the strength of their mutual interactions. A higher value of $\ln\gamma_{1}^{\infty }$ implies weaker interactions and lower affinity of component 1 toward component 2, and vice versa.^41^ The obtained NRTL model binary interaction parameters (*g*_12_, *g*_21_) and calculated infinite dilution activity coefficients ( $\ln\gamma_{1}^{\infty }$ and $\ln\gamma_{2}^{\infty }$ ) of components in the binary systems at 298.1 K are shown in Table 2. The HCT–water binary system exhibits the strongest positive deviations from ideality, as reflected in large $\ln\gamma_{1}^{\infty }$ and $\ln\gamma_{2}^{\infty }$ values for both components, indicating mutual immiscibility. Conversely, the HCT–Nic system shows strongly negative $\ln\gamma_{1}^{\infty }$ and $\ln\gamma_{2}^{\infty }$ values, indicating favorable interactions between HCT and Nic, consistent with the formation of a stable cocrystal. As shown in our previous work,^41^ Nic–water binary system shows moderately favorable interactions.

In Figure 7, the modeled SLE phase diagram of the HCT–Nic system using the NRTL model, the fitted binary interaction parameters (Table 2) and the melting properties of HCT, Nic, and their 1:1 cocrystal (Table 1) is presented. For comparison, the HCT–Nic SLE was also calculated assuming ideal solution behavior ( $\gamma_{i}^{L}$ = 1). As shown in Figure 7, the NRTL model provides a good description of the binary SLE phase diagram of the HCT–Nic system. It successfully captures the system’s nonideality and the incongruent melting dissolution behavior of the cocrystal. In contrast, assuming ideal solution behavior does not predict the cocrystal formation and instead reduces the phase diagram to that of a simple eutectic system.

**Figure 7:**
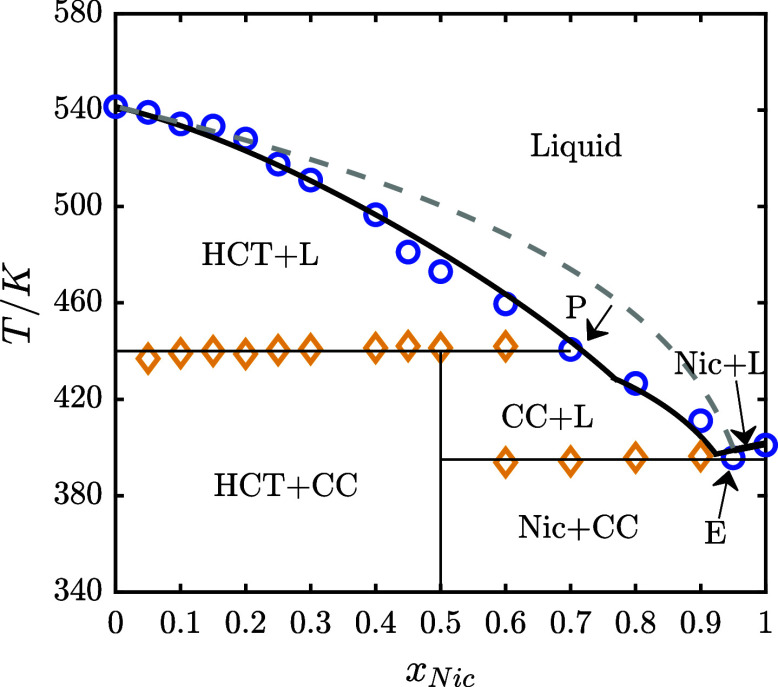
SLE phase diagrams of the HCT–Nic system. Blue circles: experimentally determined liquidus temperatures; orange diamonds: eutectic (solidus) temperatures; solid black line: NRTL-modeled liquidus; dashed gray line: ideal liquidus line.

#### 3.2.2 Modeling the SLE Phase Diagram of the Ternary Systems

The SLE in the ternary HCT–Nic–water system was modeled at 310.15 K (approximate human body temperature), 330.15 K, and 350.15 K using eq 2 and the NRTL model. The resulting phase diagrams are presented in Figures 8–10, respectively. Each figure includes: (a) the full ternary diagram, (b) a zoomed-in view of the dilute aqueous region (water-rich region), and (c) a further zoom to clearly visualize the calculated liquidus points using eq 14 (blue circles) and the NRTL-modeled solubility lines. Due to its small size, the cocrystal phase region is not visible in the full SLE phase diagrams.

**Figure 8:**
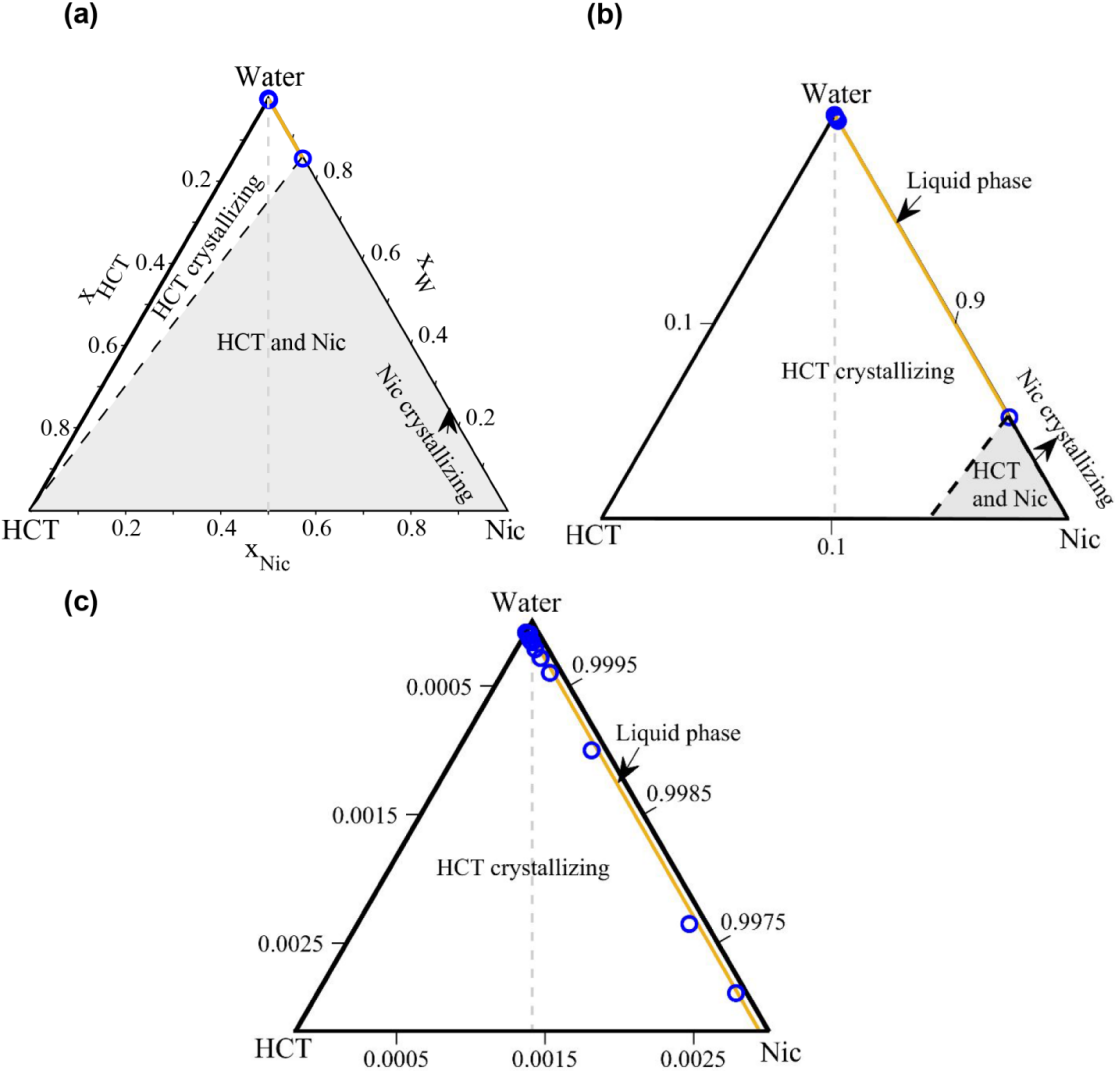
SLE phase diagrams of the HCT–Nic–water system at 310.15 K. (a) Full diagram; (b) Zoomed-in view of the water-rich region; (c) Further zoom of (b). Blue circles: experimentally calculated liquidus temperatures with eq 14, and A and B parameters from Table S3 in Supporting Information; solid orange line: NRTL-modeled HCT liquidus.

**Figure 9:**
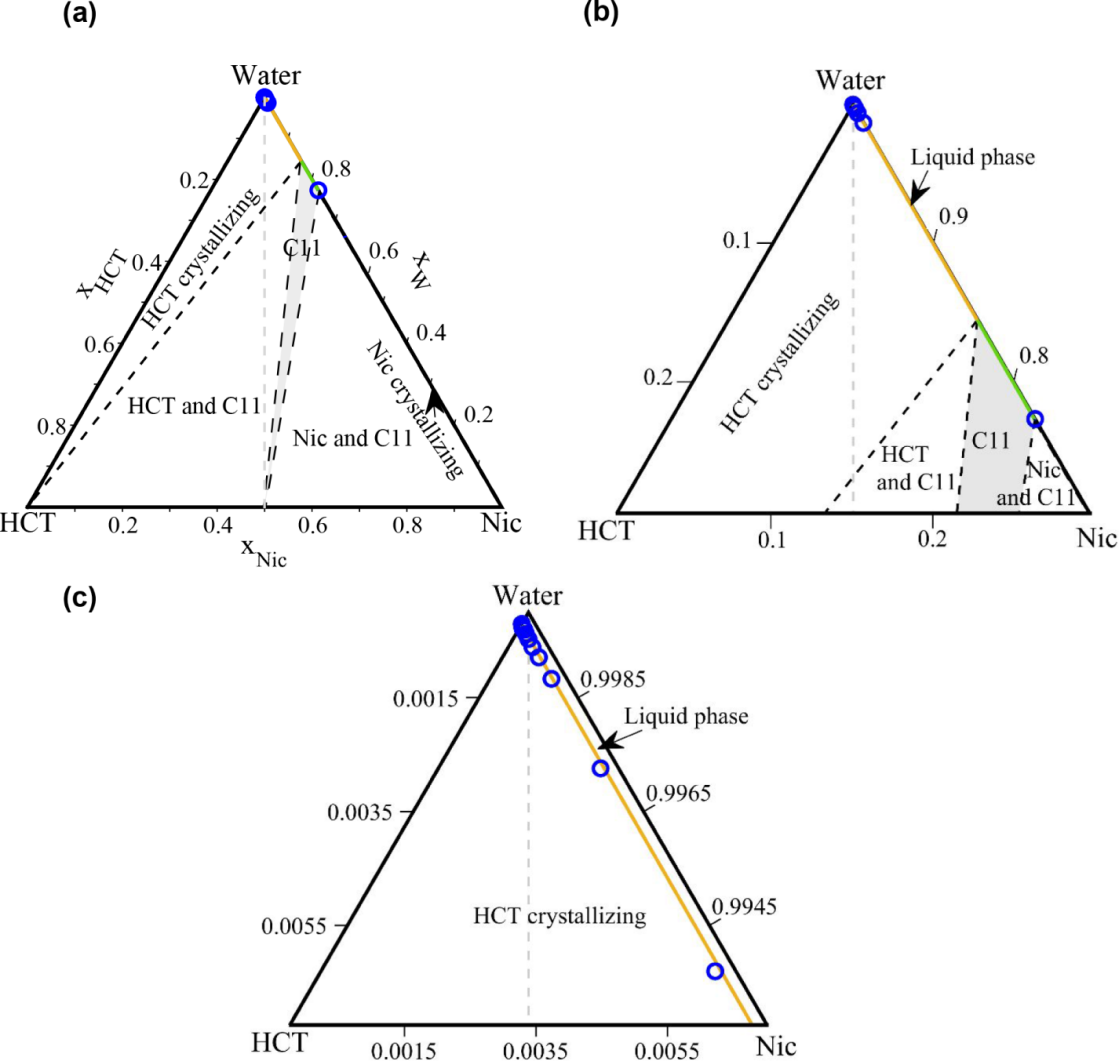
SLE phase diagrams of the HCT–Nic–water system at 330.15 K. (a) Full diagram; (b) Zoomed-in view of the water-rich region; (c) Further zoom of (b). Blue circles: experimentally calculated liquidus temperatures with eq 14, and A and B parameters from Table S3 in Supporting Information; solid orange line: NRTL-modeled HCT liquidus; solid green line: NRTL-modeled HCT–Nic cocrystal liquidus.

**Figure 10:**
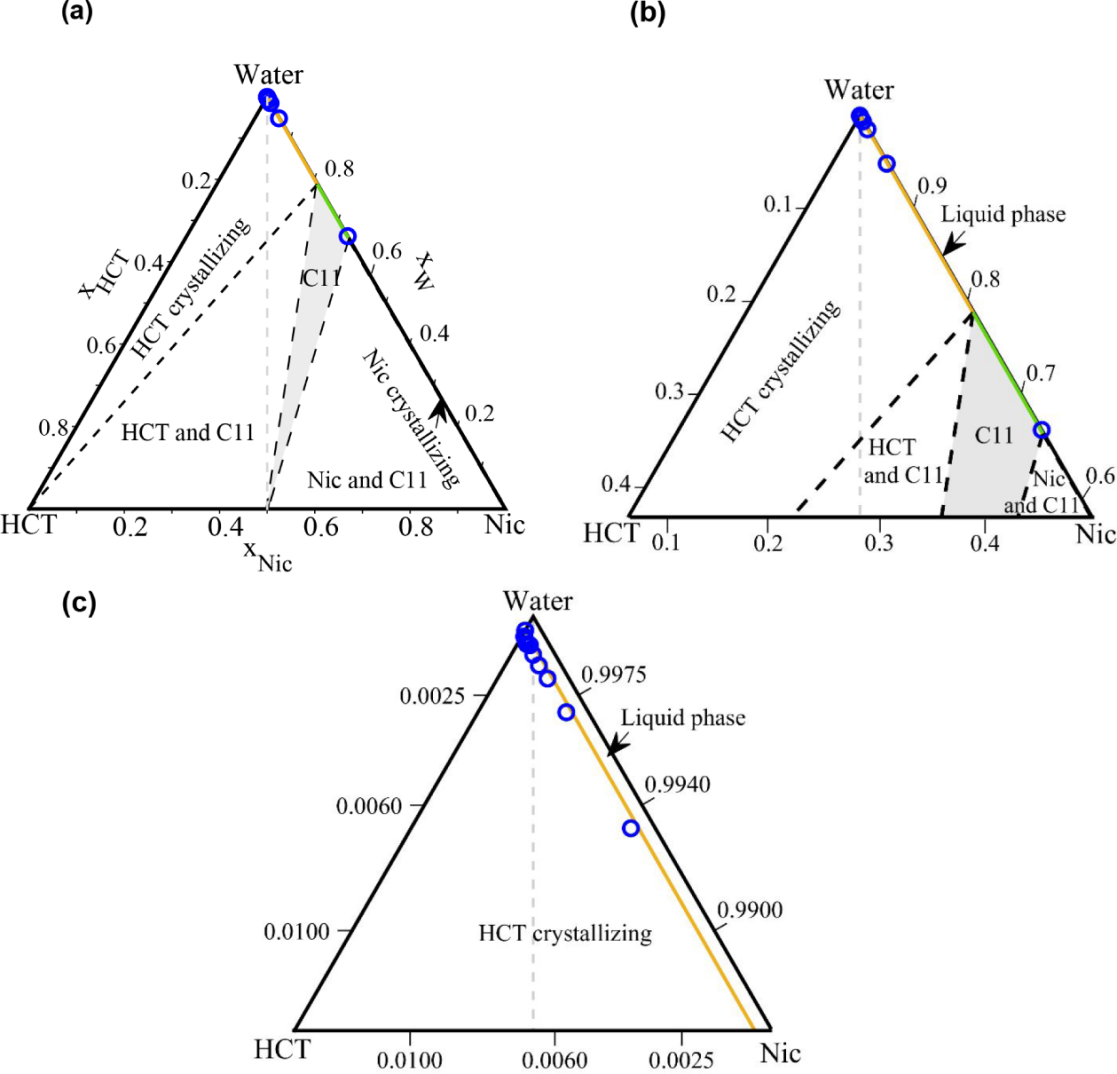
SLE phase diagrams of the HCT–Nic–water system at 350.15 K. (a) Full diagram; (b) Zoomed-in view of the water-rich region; (c) Further zoom of (b). Blue circles: experimentally calculated liquidus temperatures with eq 14, and A and B parameters from Table S3 in Supporting Information; solid orange line: NRTL-modeled HCT liquidus; solid green line: NRTL-modeled HCT–Nic cocrystal liquidus.

At 310.15 K (Figure 8), the HCT–Nic–water system exhibits eutectic behavior. The modeled isotherm consists of two solubility lines-one for HCT and one for Nic—that intersect at a eutectic point, where the API solubility reaches its maximum, as discussed in our previous works.^41,50^ Due to the significantly lower solubility of HCT compared to Nic, the HCT liquidus line spans a much broader compositional region of the ternary phase diagram than that of Nic. The NRTL-modeled liquidus line (orange line) shows excellent agreement with the experimentally calculated liquidus temperatures using eq 14 (blue circles, Figure 8c). The absence of the 1:1 cocrystal solid phase at this temperature is likely due to the significant gap between the solution temperature (310.15 K) and the melting temperature of the cocrystal (473.72 K), making cocrystal precipitation thermodynamically unfavorable.

At 330.15 K, the SLE diagram reveals a 1:1 cocrystal solid-phase region (marked with C11 in Figure 9) exhibiting incongruent dissolution behavior: the cocrystal is thermodynamically stable, yet it cannot crystallize along the 1:1 stoichiometric line (dashed gray line in Figure 9) due to the large solubility difference between HCT and Nic in water. In Figures 9 and 10, the orange line represents the HCT liquidus line, while the green line corresponds to the cocrystal solubility line. At 350.15 K (Figure 10), the cocrystal region expands further toward the 1:1 stoichiometric line; nevertheless, the cocrystal dissolves incongruently.

#### 3.2.3 Solubility Enhancement of HCT in Ternary Systems

This section illustrates how ternary API–coformer–water SLE phase diagrams can be utilized to determine the API–coformer molar ratio and water amount required to achieve a targeted API solubility at a given temperature. While the API–coformer cocrystal is often employed in the literature to enhance API solubility through direct dissolution in water, our previous studies^41,50^ on ternary API–excipient–water systems forming simple eutectic mixtures (without cocrystal formation) have demonstrated that the highest API solubility occurs at the eutectic composition in the ternary mixture. A similar observation was also confirmed in our previous study^34^ on ternary API–coformer–solvent systems, where the API and coformer form a congruently and incongruently melting cocrystals. In the present study, the solubility enhancement of HCT in the HCT–Nic–water system was evaluated in three cases: (i) HCT solubility in pure water (*x_Nic_* = 0), (ii) solubility at the composition corresponding to stoichiometric dilution of the incongruently melting cocrystal (*x_Nic_*:*x_HCT_* = 1:1) in water, and (iii) solubility at the eutectic point of the ternary system. Each case was analyzed at 310.15, 330.15, and 350.15 K (Figure 11a–c).

**Figure 11:**
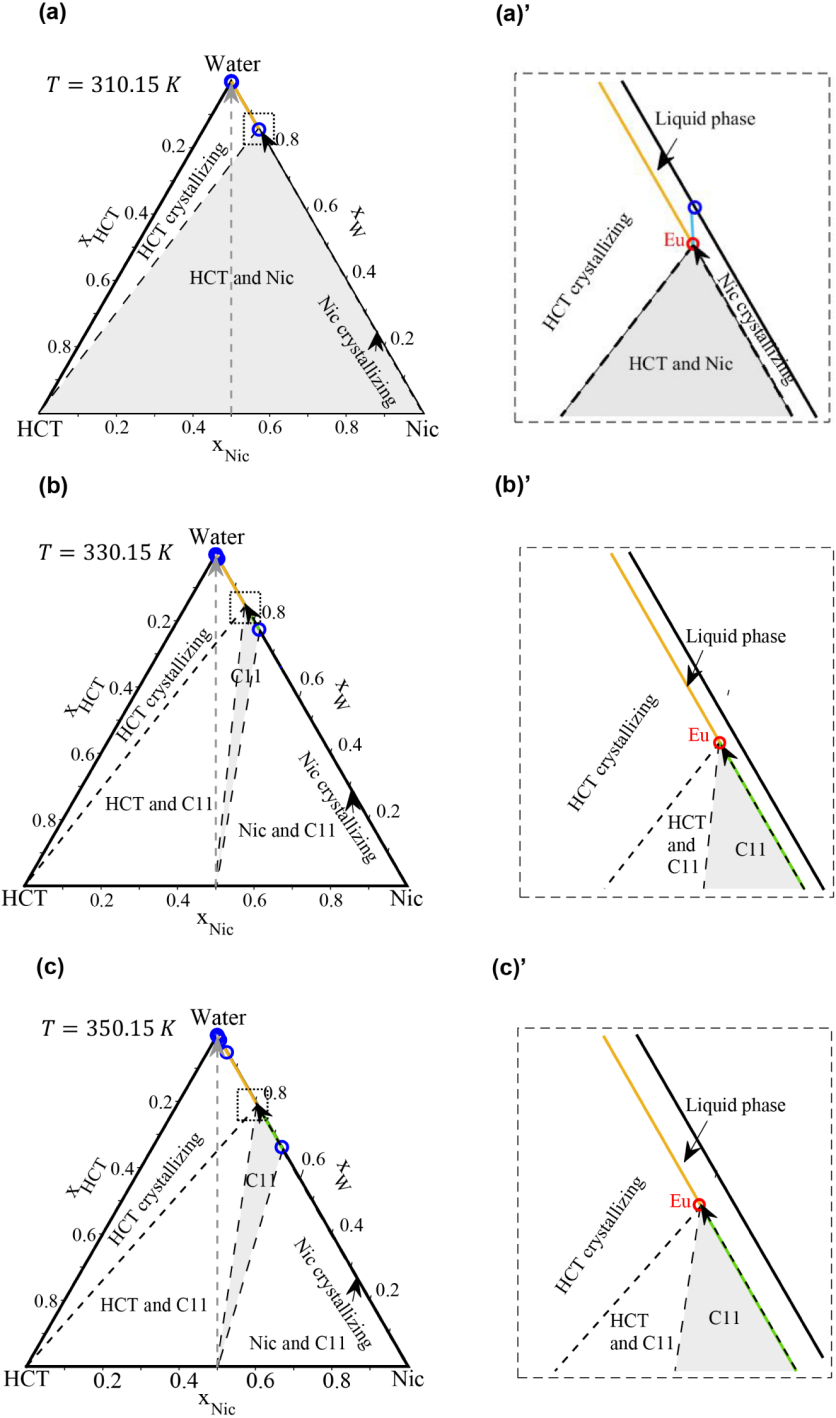
SLE phase diagrams of the HCT–Nic–water system at (a) 310.15 K; (b) 330 K; (c) 350 K. Blue circles: experimentally determined liquidus temperatures; solid orange line: NRTL-modeled HCT liquidus; solid green line: NRTL-modeled HCT–Nic cocrystal liquidus; solid light blue line: NRTL-modeled Nic liquidus; (a′-c′) Zoom-in view of the API-rich side eutectic region.

At 310.15 K (Figure 11a), the ternary HCT–Nic–water system forms a simple eutectic mixture without cocrystal formation, exhibiting a single eutectic point (Eu) defined by the intersection of the HCT and Nic liquidus lines (Figure 11a′). At higher temperatures (330.15 and 350.15 K; Figure 11b–c), incongruently melting cocrystal formation becomes thermodynamically favored, leading to the appearance of two eutectic points: one on the HCT-rich side (intersection of the HCT and cocrystal liquidus lines) and another on the Nic-rich side (intersection of the Nic and cocrystal liquidus lines). The HCT-rich eutectic point—highlighted in Figure 11b′–c′—was selected for solubility evaluation, as it corresponds to the composition with higher HCT solubility relative to the Nic-rich eutectic region. As shown in Figure 11a–c, the dashed gray lines indicate stoichiometric dilution paths originating from the HCT–Nic 1:1 cocrystal composition toward the water apex, while the dashed black arrows denote dilution paths leading to the eutectic composition in the ternary solutions at each selected temperature. In ternary API–coformer–water systems, the isotherm shape changes with temperature, resulting in different eutectic positions and, consequently, different corresponding starting API–coformer compositions.

In Figure 12a, the variation of the HCT mole fraction relative to water mole fraction (*x*_1_/*x*_3_ = *x_HCT_*/*x_W_*) is shown for the three studied cases (i–iii), calculated using eq 12 at 310.15, 330.15, and 350.15 K. For each case, the calculated *(x_HCT_*/*x_W_*) values increase with temperature, and the highest values are consistently observed at the eutectic composition in the ternary solution. The corresponding HCT solubility enhancement factors (Φ), calculated using eq 13, are presented in Figure 12b. The maximum Φ values (Φ ≈ 2.1–2.4) are observed at the ternary eutectic point across all studied temperatures (310.15, 330.15, and 350.15 K). Interestingly, at 310.15 K, where the ternary HCT–Nic–water system exhibits eutectic behavior—although this temperature is lower than those at which the system forms a cocrystal (330.15 and 350.15 K)—the solubility enhancement factor (Φ) at the eutectic composition is the highest (orange line in Figure 12b). These findings indicate that dilution of the HCT–Nic 1:1 molar ratio cocrystal in water does not yield the highest HCT solubility. Instead, starting from a solid HCT–Nic mixture composition that, upon dilution, results in an aqueous solution corresponding to the eutectic composition in the ternary phase diagram is more effective in achieving the maximum possible enhancement of HCT solubility at any temperature.

**Figure 12:**
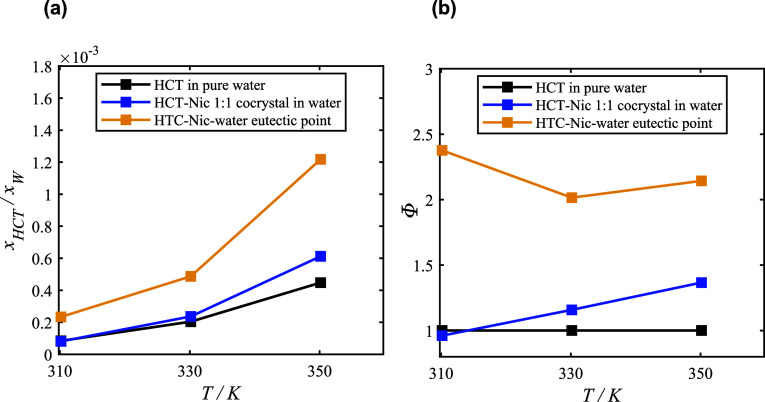
(a) *x_HCT_*/*x_W_* as a function of the solution temperature. Black line: HCT solubility in pure water; blue line: HCT solubility obtained with HCT–Nic 1:1 cocrystal; solid orange line: HCT solubility at the eutectic point in the ternary HCT–Nic–water system; (b) The corresponding solubility enhancement factor (Φ).

## 4 Conclusions

This study aimed to understand the aqueous solubility enhancement of APIs that form incongruently melting cocrystals from a thermodynamic perspective, using HCT–Nic as a case study. A combined experimental and modeling approach was employed to evaluate the solubility behavior and phase equilibria of the HCT–Nic–water system. The SLE data of the subbinary systems (HCT–Nic, HCT–water, and Nic–water) were measured at different temperatures using temperature-variant methods. These data, along with the melting properties of the components, were used to fit NRTL model binary interaction parameters. The resulting parameters were then used to model the ternary SLE phase diagram of the HCT–Nic–water system at different temperatures. The findings revealed that the HCT–Nic–water system behaves as a eutectic system at 310.15 K (a temperature well below the cocrystal melting point), with no cocrystal formation. At higher temperatures (330.15 K and 350.15 K), the HCT–Nic cocrystal becomes thermodynamically stable in the ternary system, exhibiting incongruent dissolution behavior. Importantly, HCT solubility at each studied temperature was found to be highest at the eutectic composition on the API-rich side of the ternary HCT–Nic–water system, and that selecting a binary HCT:Nic molar composition which, upon dilution, results in the eutectic composition is more effective for solubility enhancement than starting from the cocrystal composition with subsequent dilution with water. At 310.15 K, where the HCT–Nic–water system forms a simple eutectic without cocrystal formation, the solubility enhancement factor (Φ) is higher than at the corresponding eutectic compositions (API-rich region) at 330.15 and 350.15 K. Overall, this work deepens the thermodynamic understanding of incongruently melting pharmaceutical cocrystal systems and supports their rational design and application in pharmaceutical development. The proposed approach and the findings are not limited to the HCT–Nic–water system but can also serve as a framework for investigating other incongruently melting API–coformer systems.

## Supplementary Material



## Data Availability

The data supporting this article have been included in the main manuscript and the Supporting Information file.

## Notes

The PXRD patterns of pure HCT and Nic at ambient temperature, compared to calculated patterns; the PXRD patterns of the HCT–Nic cocrystal, compared to pure components at different temperatures; the DSC thermograms of pure HCT, pure Nic, and the 1:1 HCT–Nic cocrystal; the binary SLE data of the HCT–Nic system; the fitted parameters for the empirical correlation of HCT solubility in water with and without nicotinamide; the measured solubility data of HCT, Nic, and their mixtures in water at different molar ratios and temperatures using the temperature-variant method (PDF)

## Notes

During the preparation of this work, the author(s) used ChatGPT to improve the readability and language of the manuscript. After using this tool, the author(s) carefully reviewed and edited the content as needed and took full responsibility for the final version of the publication.
