# Supplementary material for: Solubility of Incongruently Melting Active Pharmaceutical Ingredient Cocrystals: The Hydrochlorothiazide–Nicotinamide System
Source: Mol Pharm. 2026 Feb 10;23(3):1770–84. doi: 10.1021/acs.molpharmaceut.5c01520 (PMC12958295; doi:10.1021/acs.molpharmaceut.5c01520)
Supplement: Supplementary file 1 [file mp5c01520_si_001.pdf]

Solubility of Incongruently Melting Active Pharmaceutical Ingredient Cocrystals: The Hydrochlorothiazide–Nicotinamide System

Sahar Nasrallah, Tejas Gavali, Isil Yavuz, and Mirjana Minceva*

Biothermodynamics, TUM School of Life Sciences, Technical University of Munich, Maximus-von Imhof-Forum 2, Freising 85354, Germany

*Corresponding author e-mail: mirjana.minceva@tum.de

Powder X-ray diffraction

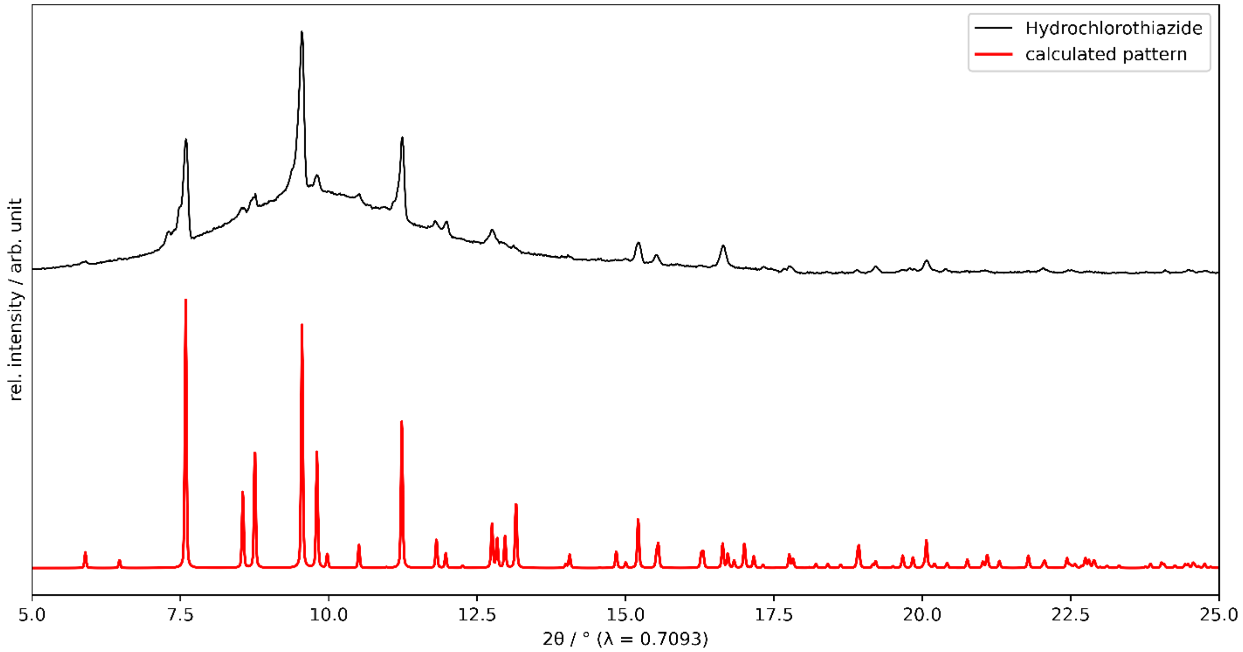

Figure S1. The PXRD pattern of the pure hydrochlorothiazide (HCT) sample (black) at ambient temperature compared to the calculated pattern from single-crystal X-ray diffraction (red) at 298 K.[1]

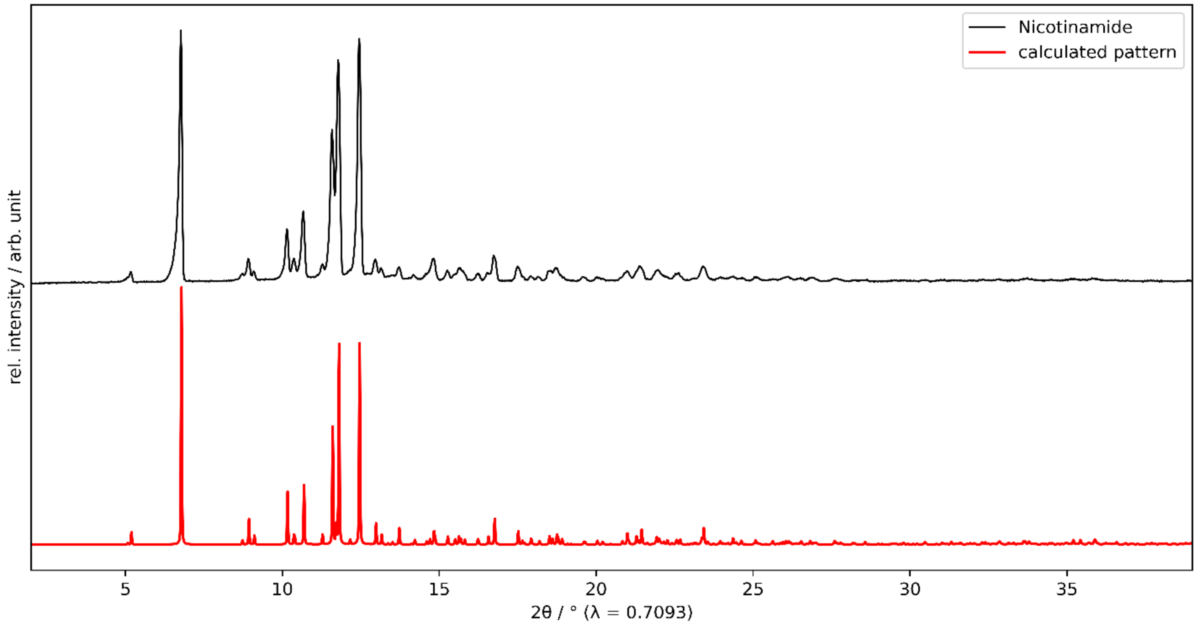

Figure S2. The PXRD pattern of the pure nicotinamide (Nic) sample (black) at ambient temperature compared to the calculated pattern from single-crystal X-ray diffraction (red) at 295 K.[2]

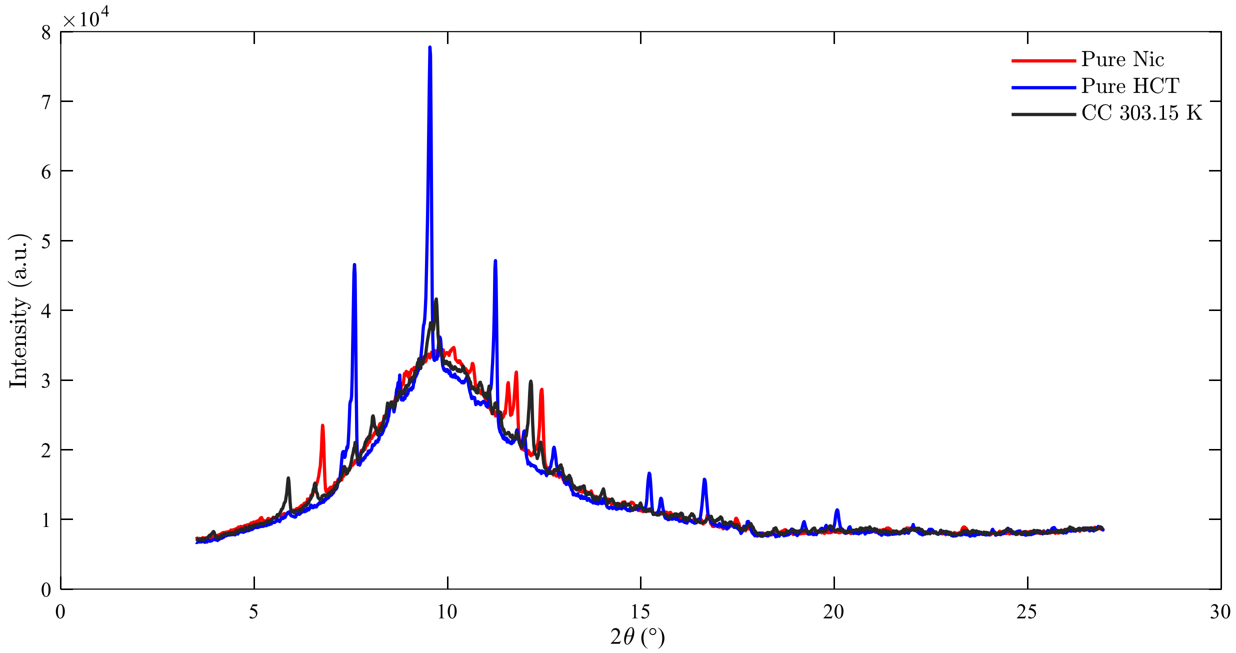

Figure S3. PXRD pattern of the HCT–Nic cocrystal (CC, 1:1 molar ratio, black) compared with pure HCT (blue) and pure nicotinamide (red) at 303.15 K.

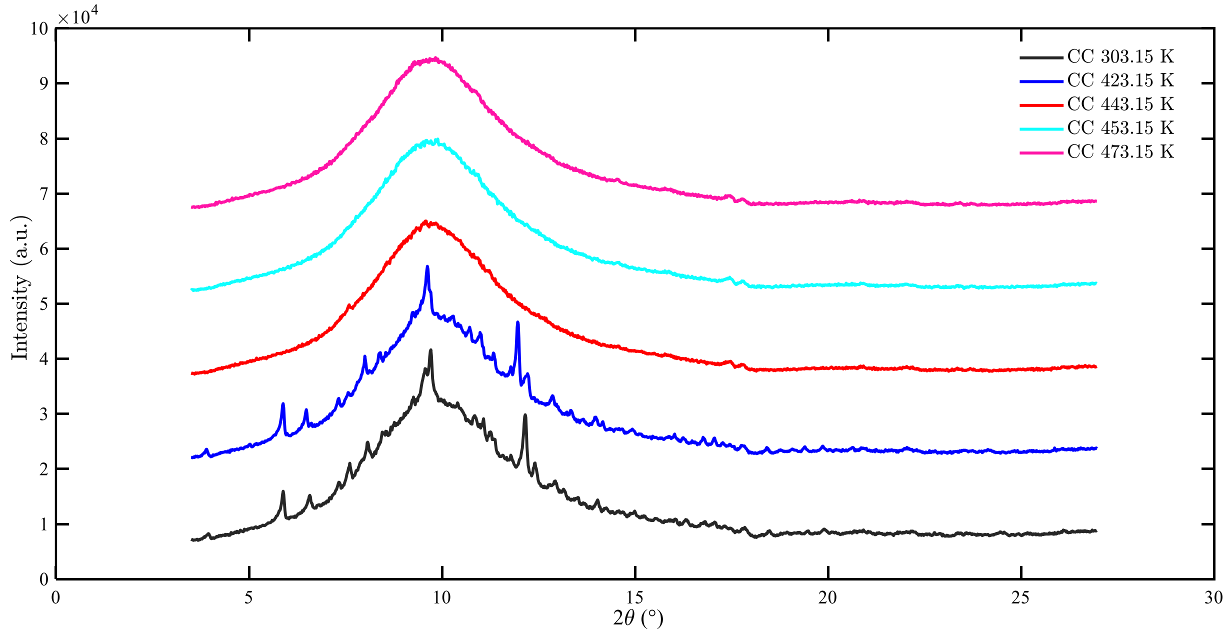

Figure S4. Stacked PXRD patterns of the HCT–Nic cocrystal (CC, 1:1 molar ratio, black) measured at various temperatures (303.15–473.15 K). At temperatures above 443.15 K, the diffraction patterns become similar due to loss of crystallinity following cocrystal decomposition.

Differential scanning calorimetry (DSC)

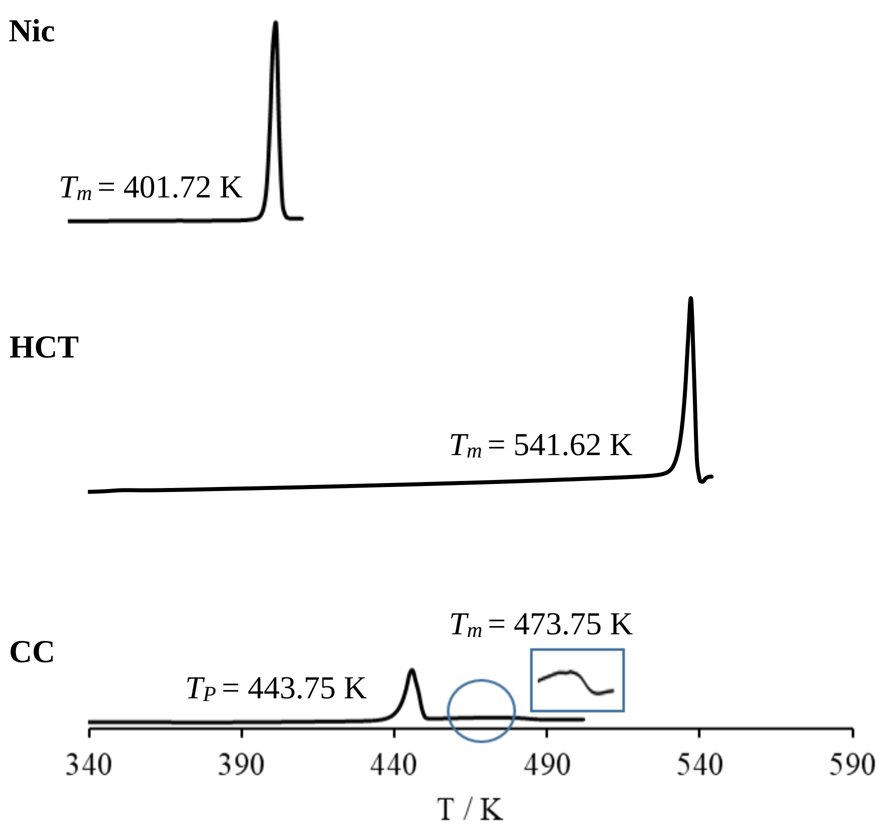

Figure S5. Differential scanning calorimetry thermograms of pure components and the HCT–Nic 1:1 cocrystal (CC).

Table S1. Solid–liquid equilibria data of HCT–Nic binary system.

x_{Nic}	T_E/K	T_P/K	T^{liq}/K
0.95	396.05 ± 0.10	-	396.05 ± 0.10
0.90	396.55 ± 0.06	-	411.05 ± 0.70
0.80	395.95 ± 0.10	-	426.75 ± 0.10
0.70	394.35 ± 0.23	-	440.75 ± 0.38
0.60	393.95 ± 0.32	442.15 ± 0.11	446.85 ± 0.15
0.50	-	443.75 ± 0.06	473.75 ± 0.76
0.40	-	441.45 ± 0.15	494.75 ± 0.72
0.30	-	440.95 ± 0.46	510.85 ± 0.12
0.20	-	438.95 ± 0.21	532.95 ± 0.20
0.10	-	439.25 ± 0.40	533.25 ± 1.11

The solubility data of HCT–Nic mixtures in water

Table S2. Measured solubility values of the HCT–Nic mixtures in water using the temperature variant method (n=3).

x_{HCT}	**T/K**	**1/T (K^{-1})**	lnx_{HCT}
HCT: Nic (0.025:0.975)			
0.00007	309.46 ± 0.27	0.00323	-9.55149
0.00009	312.79 ± 0.17	0.00319	-9.32915
0.00012	317.53 ± 0.40	0.00315	-8.99018
0.00016	320.88 ± 0.09	0.00312	-8.7435
0.00021	323.29 ± 0.25	0.00309	-8.45804
0.00026	326.50 ± 0.30	0.00306	-8.23727
HCT: Nic (0.05:0.95)			
0.00005	293.62 ± 0.19	0.00341	-9.93946
0.00007	301.99 ± 0.53	0.00331	-9.57809
0.00009	304.22 ± 0.47	0.00329	-9.32285
0.00012	309.94 ± 0.58	0.00323	-9.05372
0.00014	312.33 ± 0.33	0.00320	-8.88963
0.00017	318.19 ± 0.88	0.00314	-8.66757
0.00021	322.75 ± 0.22	0.00309	-8.48634
0.00027	326.92 ± 0.33	0.00306	-8.20272
0.00031	328.06 ± 0.37	0.00305	-8.08453
HCT: Nic (0.1:0.9)			
0.00004	294.77 ± 0.84	0.00339	-10.1632

0.00006	300.62 ± 0.17	0.00333	-9.65189
0.00009	307.46 ± 0.31	0.00325	-9.31457
0.00013	315.86 ± 0.86	0.00317	-8.95888
HCT: Nic (0.2:0.8)			
0.00007	306.70 ± 0.35	0.00326	-9.58829
0.00010	315.54 ± 0.34	0.00317	-9.17639
0.00016	323.43 ± 0.42	0.00309	-8.73375
0.00027	333.68 ± 0.62	0.00299	-8.20156
0.00039	339.31 ± 0.14	0.00295	-7.85211
HCT: Nic (0.3:0.7)			
0.00009	311.17 ± 0.48	0.00321	-9.28280
0.00022	329.41 ± 0.62	0.00303	-8.43742
0.00031	337.70 ± 0.98	0.00296	-8.08003
0.00046	341.62 ± 0.52	0.00292	-7.67642
HCT: Nic (0.4:0.6)			
0.00004	296.90 ± 0.92	0.00337	-10.1893
0.00011	314.40 ± 0.63	0.00318	-9.09560
0.00019	324.34 ± 0.96	0.00308	-8.57878
0.00026	329.93 ± 0.49	0.00303	-8.24636
0.00037	340.08 ± 0.51	0.00294	-7.88899
0.00045	343.38 ± 0.26	0.00291	-7.71132
HCT: Nic (0.5:0.5)			

0.00003	293.00 ± 0.54	0.00341	-10.4143
0.00004	299.18 ± 0.19	0.00334	-10.0581
0.00006	305.81 ± 0.62	0.00326	-9.72230
0.00007	308.09 ± 0.20	0.00324	-9.58888
0.00009	313.05 ± 0.65	0.00319	-9.36498
0.00013	318.95 ± 0.38	0.00313	-8.96139
0.00017	321.50 ± 0.82	0.00311	-8.67521
0.00022	325.23 ± 0.32	0.00307	-8.44275
0.00026	333.35 ± 0.14	0.00299	-8.25567
0.00030	335.83 ± 0.18	0.00298	-8.11025
HCT: Nic (0.6:0.4)			
0.00002	287.68 ± 0.81	0.00348	-10.6254
0.00005	300.38 ± 0.64	0.00333	-9.94431
0.00007	308.30 ± 0.54	0.00324	-9.52933
0.00009	311.78 ± 0.45	0.00321	-9.27034
0.00012	315.27 ± 0.21	0.00317	-9.02275
0.00014	321.40 ± 0.21	0.00311	-8.85128
0.00019	326.10 ± 0.56	0.00307	-8.56701
0.00021	328.75 ± 0.91	0.00304	-8.44552
0.00026	332.75 ± 0.70	0.00301	-8.24323
0.00031	337.02 ± 1.07	0.00297	-8.08199
HCT: Nic (0.7:0.3)			

0.00003	296.63 ± 0.45	0.00337	-10.5453
0.00005	307.23 ± 0.83	0.00326	-9.87446
0.00010	316.22 ± 0.36	0.00316	-9.18239
0.00013	320.69 ± 0.73	0.00312	-8.95259
0.00016	323.94 ± 0.20	0.00309	-8.76236
0.00018	325.59 ± 0.98	0.00307	-8.62119
0.00020	329.73 ± 0.02	0.00303	-8.49432
0.00026	333.43 ± 0.30	0.00299	-8.26642
0.00031	338.17 ± 0.77	0.00296	-8.08171
HCT: Nic (0.8:0.2)			
0.00006	303.82 ± 0.40	0.00329	-9.79104
0.00008	312.02 ± 0.75	0.00321	-9.3792
0.00011	315.38 ± 0.48	0.00317	-9.10497
0.00014	318.29 ± 0.59	0.00314	-8.88816
0.00017	322.36 ± 0.20	0.00310	-8.70744
0.00019	326.47 ± 0.43	0.00306	-8.54682
0.00025	331.30 ± 0.23	0.00302	-8.30591
0.00030	334.10 ± 0.91	0.00299	-8.10139
0.00036	341.05 ± 0.74	0.00293	-7.93059
HCT: Nic (0.9:0.1)			
0.00003	295.03 ± 0.23	0.00339	-10.4073
0.00006	306.67 ± 0.58	0.00326	-9.72593

0.00009	310.47 ± 0.59	0.00322	-9.3409
0.00012	316.45 ± 0.84	0.00316	-9.04209
0.00014	321.22 ± 0.58	0.00311	-8.84169
0.00017	324.89 ± 0.45	0.00307	-8.65448
0.00023	330.19 ± 0.57	0.00303	-8.36888
0.00026	334.09 ± 0.75	0.00299	-8.2516
0.00032	338.84 ± 0.14	0.00295	-8.05043
Pure HCT			
0.000037	292.81 ± 0.52	0.00342	-10.2046
0.000043	298.30 ± 1.14	0.00335	-10.0543
0.00005	300.19 ± 0.24	0.00333	-9.90349
0.000063	305.79 ± 1.09	0.00327	-9.67237
0.000076	308.21 ± 0.86	0.00324	-9.48477
0.000096	312.72 ± 0.95	0.00319	-9.25116
0.000116	317.86 ± 1.03	0.00315	-9.06192
0.000138	320.28 ± 0.49	0.00312	-8.88825
0.000151	323.47 ± 0.78	0.00309	-8.79823
0.000182	327.27 ± 0.56	0.00306	-8.61150
Pure Nic			
x_{Nic}	**T/K**	**1/T (K^{-1})**	lnx_{Nic}
0.098739	297.10 ± 0.07	0.00337	-2.31527
0.114450	301.05 ± 0.56	0.00332	-2.16761

0.133013	305.65 ± 0.71	0.00327	-2.01730
0.153171	310.85 ± 0.28	0.00323	-1.87619
0.169682	314.60 ± 0.35	0.00318	-1.77382
0.196244	324.18 ± 0.66	0.00308	-1.62839
0.248683	335.97 ± 1.04	0.00298	-1.39157
0.297089	343.28 ± 0.89	0.00291	-1.21372

Table S3. Fitted A and B parameters of the empirical solubility correlation Eq.14 for the HCT-Nic-water system at various HCT:Nic molar ratios. The correlation coefficients (R^2) indicate excellent fit quality across all compositions.

$x_{HCT} : x_{Nic}$	$A\ (T^{-1}) + B$	R^2
0:1 (pure Nic)	-2309.3 (T^{-1}) + 5.5137	0.9904
0.025:0.975	-7874.8 (T^{-1}) + 15.855	0.9923
0.05:0.95	-5086.1 (T^{-1}) + 7.3506	0.9921
0.1:0.9	-5196.3 (T^{-1}) + 7.5444	0.9764
0.2:0.8	-5539.9 (T^{-1}) + 8.4253	0.9957
0.3:0.7	-5292.4 (T^{-1}) + 7.6904	0.9783
0.4:0.6	-5404.7 (T^{-1}) + 8.0603	0.9967
0.5:0.5	-5493 (T^{-1}) + 8.2884	0.9884
0.6:0.4	-5074.6 (T^{-1}) + 6.9878	0.9972
0.7:0.3	-6078.5 (T^{-1}) + 9.9717	0.9952
0.8:0.2	-5359.4 (T^{-1}) + 7.8743	0.9909
0.9:0.1	-5396.9 (T^{-1}) + 7.9426	0.9934
1:0 (pure HCT)	-4618.1 (T^{-1}) + 5.4896	0.9940

REFERENCES

(1) Dupont, L.; Dideberg, O. Structure cristalline de l'hydrochlorothiazide, C7H8ClN3O4S2. *Structural Science* **1972**, *28* (8), 2340-2347.

(2) Wright, W.; King, G. The crystal structure of nicotinamide. *Acta Crystallographica* **1954**, *7* (3), 283-288.
